# Conformation-dependent dynamic organic phosphorescence through thermal energy driven molecular rotations

**DOI:** 10.1038/s41467-023-35930-5

**Published:** 2023-02-06

**Authors:** Juan Wei, Chenyuan Liu, Jiayu Duan, Aiwen Shao, Jinlu Li, Jiangang Li, Wenjie Gu, Zixian Li, Shujuan Liu, Yun Ma, Wei Huang, Qiang Zhao

**Affiliations:** 1 State Key Laboratory of Organic Electronics and Information Displays & Jiangsu Key Laboratory for Biosensors, Institute of Advanced Materials (IAM) & Institute of Flexible Electronics (Future Technology), Nanjing University of Posts and Telecommunications (NUPT), Nanjing, 210023 P. R. China; 2 Frontiers Science Center for Flexible Electronics (FSCFE), MIIT Key Laboratory of Flexible Electronics (KLFE), Northwestern Polytechnical University, Xi’an, 710072 P.R. China; 3 College of Electronic and Optical Engineering & College of Flexible Electronics (Future Technology), Jiangsu Province Engineering Research Center for Fabrication and Application of Special Optical Fiber Materials and Devices, Nanjing University of Posts and Telecommunications (NUPT), 9 Wenyuan Road, Nanjing, 210023 P.R. China

**Keywords:** Polymers, Optical materials, Synthesis and processing

## Abstract

Organic room-temperature phosphorescent (RTP) materials exhibiting reversible changes in optical properties upon exposure to external stimuli have shown great potential in diverse optoelectronic fields. Particularly, dynamic manipulation of response behaviors for such materials is of fundamental significance, but it remains a formidable challenge. Herein, a series of RTP polymers were prepared by incorporating phosphorescent rotors into polymer backbone, and these materials show color-tunable persistent luminescence upon excitation at different wavelengths. Experimental results and theoretical calculations revealed that the various molecular conformations of monomers are responsible for the excitation wavelength-dependent (Ex-De) RTP behavior. Impressively, after gaining insights into the underlying mechanism, dynamic control of Ex-De RTP behavior was achieved through thermal energy driven molecular rotations of monomers. Eventually, we demonstrate the practical applications of these amorphous polymers in anti-counterfeiting areas. These findings open new opportunities for the control of response behaviors of smart-responsive RTP materials through external stimuli rather than conventional covalent modification method.

## Abstract (web summary)

Organic room-temperature phosphorescent (RTP) materials exhibiting stimuli responsive reversible changes in optical properties show great potential in diverse optoelectronic fields but a lack of mechanistic understanding limits their development. Here, the authors prepare a series of RTP polymers by incorporating phosphorescent rotors into polymer backbones and demonstrate colortunable persistent luminescence upon excitation at different wavelengths

## Introduction

Recently, organic persistent room-temperature phosphorescent (RTP) materials have emerged as critically notable materials for diverse optoelectronic applications, such as bioimaging^1–3^, chemical sensing^4,5^, optical data storage^6,7^, and anti-counterfeiting^8,9^. So far, various groundbreaking strategies related to organic persistent RTP materials have been demonstrated, many of which have displayed different phosphorescence colors, high quantum efficiencies, and long emission decay times^10–14^. In particular, smart-responsive persistent RTP materials manipulated by external stimuli have attracted considerable interest because the ability to dynamically control persistent RTP properties can endow these materials with revolutionary performance for various applications^15–17^. Towards this end, numerous stimuli-responsive persistent RTP materials exhibiting emission intensity, color, and lifetime switching have been developed^18–21^. Nevertheless, the response behaviors of them are difficult to manipulate because of the lack of understanding of underlying mechanism. In fact, controlling the response behaviors of stimuli-responsive RTP materials is quite important to meet the criteria for various optoelectronic applications. For example, modulating the photoactivation speeds and emission decay times of photoactivated organic persistent RTP molecules is crucial to achieve high-level information encryption^22,23^. The regulation of persistent RTP colors of excitation-wavelength-dependent (Ex-De) materials is beneficial to realizing multicolor displays^24,25^. However, complex chemical synthesis is often required to achieve the regulation of response behaviors, making the manipulation process inconvenient and inefficient.

**Fig. 1:**
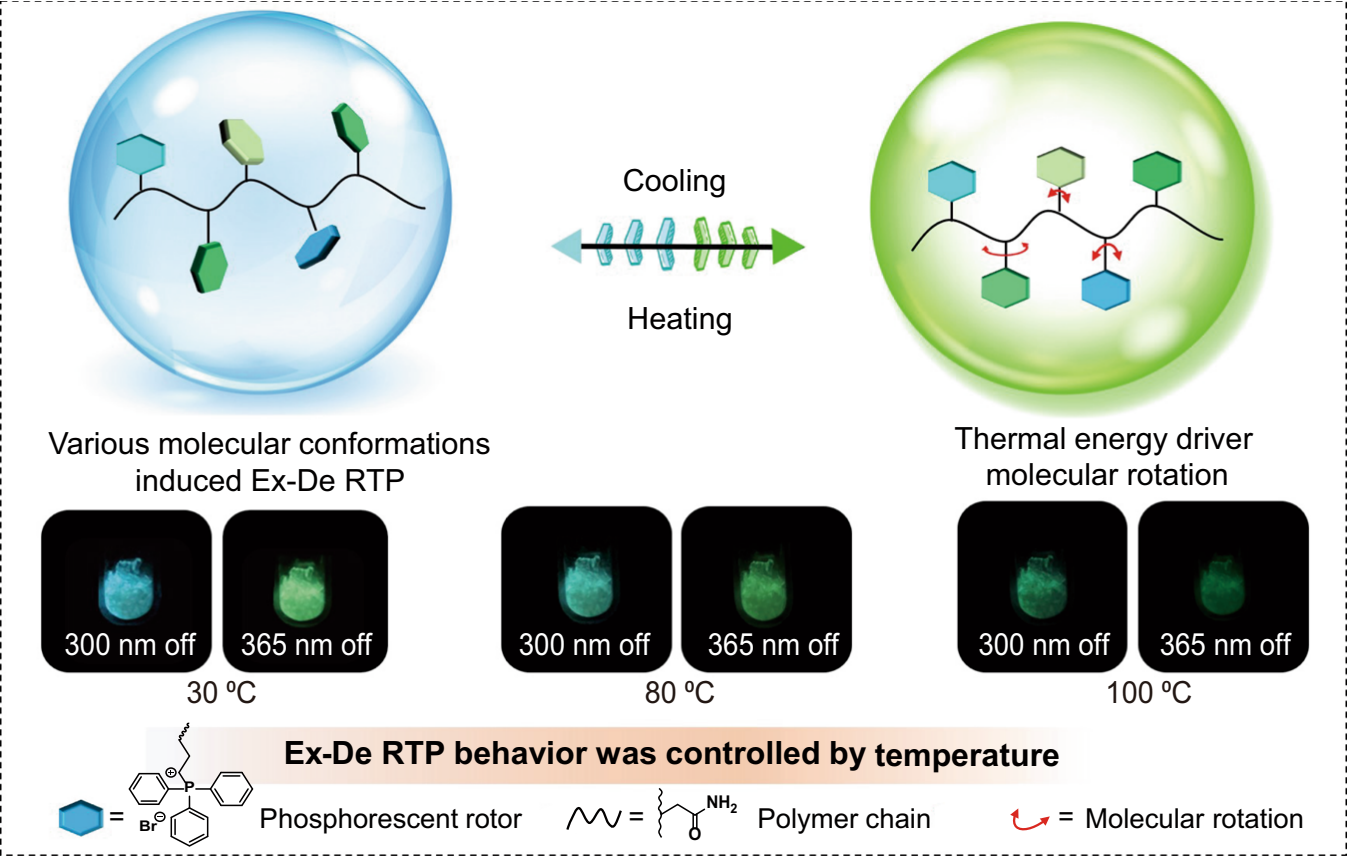
Schematic representation for the dynamic manipulation of Ex-De RTP behavior. The Ex-De RTP property is originated from various molecular conformations of monomers, and the Ex-De RTP behavior can be controlled by thermal energy.

It has been reported that organic quaternary phosphonium derivatives exhibit persistent RTP due to proximity-induced intermolecular electronic coupling in a rigid environment^26–28^. Of-note, aromatic groups were directly bonded to the phosphorus center in quaternary phosphonium derivatives to form a typical molecular rotor; therefore, they may adopt various conformations in rigid surroundings since the rotating aromatic groups will be fixed at different angles, which are closely related to different triplet energy levels^29–32^. This feature makes controlling persistent RTP colors possible by employing different excitation energies. In addition, the thermal energy might cause molecular rotations of quaternary phosphonium derivatives and thus result in conformational changes. On this basis, we envisage that it is possible to regulate the Ex-De RTP behavior of quaternary phosphonium derivative-based polymers by manipulation of the molecular conformations via thermal energy (Fig. 1).

## Results

### Synthesis and characterization

To validate our hypothesis, (but-3-en-1-yl)triphenylphosphonium bromide (M1) was selected as monomer to copolymerize with acrylamide in molar ratio of 1: 50 to prepare a RTP polymer (P1). The reason why acrylamide was chosen to copolymerize with M1 is because polyacrylamide can provide a hydrogen bond cross-linked network to restrict molecular motion^33^, and the water-soluble characteristic of polyacrylamide is beneficial to large-area processability in practical applications. The detailed synthetic procedures are given in the supplementary information (Supplementary Fig. 1). The obtained monomers were characterized by ^1^H nuclear magnetic resonance (NMR) spectroscopy, ^13^C NMR spectroscopy, and high-resolution mass spectrometry. The obtained polymers were characterized by gel permeation chromatography (GPC) and ^1^H NMR (Supplementary Figs. 2–27 and Supplementary Tab 1). Thermogravimetry analysis (TGA) was carried for the obtained polymers, and the results show that their decomposition temperatures are ranged from 215 °C to 300 °C (Supplementary Fig. 28), indicating their good thermal stabilities.

### Ex-De persistent RTP

**Fig. 2:**
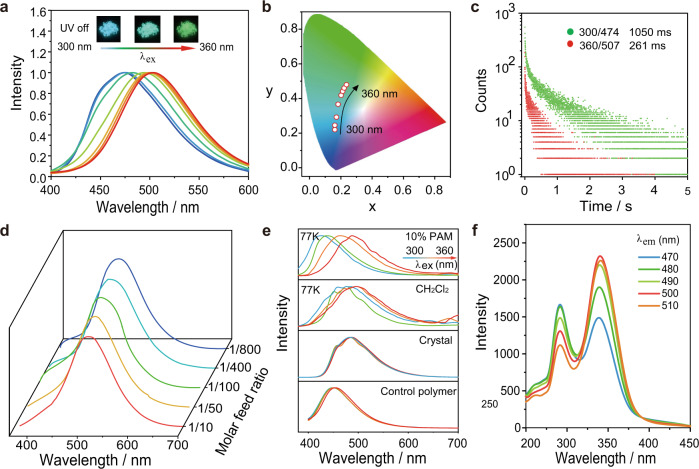
Ex-De persistent RTP and mechanism study. **a** Ex-De phosphorescence spectra of P1 upon changing excitation wavelengths. **b** CIE chromaticity diagram for P1 at various excitation wavelengths. **c** Lifetime decay curves of P1 in the solid state. **d** Phosphorescence spectra of P1 at different ratios (1/10–1/800) under 340 nm excitation. **e** Ex-De phosphorescence spectra of C1 in the crystal, CH_2_Cl_2_ (77 K), 10% PAM (77 K), and control polymer at different excitation wavelengths. **f** Excitation spectra of P1 solid monitored at various phosphorescence peaks.

The photophysical properties of P1 was studied by steady-state photoluminescence (PL), delayed PL, and time-decay spectra in the solid state. The detailed photophysical data were given in the Supplementary Figs. 29, 30 and Supplementary Tab 2. P1 presents intense absorption peaks below 350 nm, which originate from the intramolecular π-π, π-π* or n-π* transitions (Supplementary Fig. 29). Upon UV excitation, P1 has a blue fluorescence under ambient condition. As shown in Fig. 2a, sky-blue persistent luminescence was observed from P1 powder under ambient conditions after the removal of the 300 nm ultraviolet lamp. As anticipated, the afterglow colors significantly changed from sky blue to green upon changing the excitation wavelength to 360 nm. The photophysical property investigation showed that the delayed PL maxima could be adjusted from 474 to 506 nm by changing the excitation wavelength, which is consistent with the persistent RTP colors after removing the UV source. The Commission Internationale del’Eclairage (CIE) coordinates (Fig. 2b) of the persistent RTP colors corresponded to sky blue (0.18, 0.24), cyan (0.19, 0.36), and green (0.21, 0.47), which are in accordance with the naked-eye observation (Fig. 2a). Figure 2c showed that the emission decay times of P1 at 300 nm and 365 nm excitation were measured to be 1050 ms and 261 ms. In addition, the photostability of P1 in solid state has been determined. As shown in Supplementary Fig. 31, after continuous irradiation of P1 by a 300 nm UV light for 500 mins, the phosphorescence intensity at 474 nm slightly enhanced, which might be due to the consumption of oxygen under continuous UV irradiation. The effect of relative humidity (RH) on the RTP property of P1 has also been studied. The results showed that the phosphorescence intensity of P1 was significantly decreased with the increase of RHs (Supplementary Fig. 32).

### Mechanism study

Generally, anti-Kasha’s rule emissions^34,35^, isolated-aggregation emission^24,36,37^, multiemissive centers^38^, and clustering-triggered emission^18^ are used to explain Ex-De afterglow behavior. The latter two mechanisms are unlikely to explain the color-tunable afterglow of P1 because the single emissive center of monomers in the polymer and these monomers are obviously conventional luminophores. To reveal the underlying mechanism of Ex-De afterglow emission of P1, the monomer was copolymerized with acrylamide in molar ratios of 1:800, 1:400, 1:200, 1:100, and 1:10, respectively, to prepare a series of control RTP polymers. Then, their phosphorescence spectra were recorded under ambient conditions. As shown in Fig. 2d, we can see that no new emission band emerged for P1 upon increasing the monomer concentrations from 1:800 to 1:10. In addition, all polymers exhibit Ex-De RTP behaviors. The ratiometric change of two emission wavelengths was studied, and the relationship between these two bands is nonlinear (Supplementary Figs. 33 and 34). Thus, the isolated-aggregation emission mechanism might not be responsible for the Ex-De persistent RTP behavior. In addition, butyltriphenylphosphonium bromide (C1) was synthesized to conduct a series of control experiments. First, the optical property of C1 doped polyacrylamide (PAM) was studied. Compared with copolymers, the doped polymer showed less restriction than the monomer, leading to inefficient RTP behavior^33^. Thus, the delayed PL spectrum of the doped polymer film (10 wt.%) was measured at 77 K under different wavelength excitation. Figure 2e illustrates that the RTP peak at 430 nm shifted to 487 nm upon increasing the excitation wavelength, which caused the afterglow color transition from blue to cyan. Moreover, the Ex-De persistent RTP behavior from blue to cyan was clearly detected in CH_2_Cl_2_ solution at 77 K (Fig. 2e). Taken together, the isolated-aggregation emission mechanism can be excluded from accounting for the Ex-De persistent RTP behavior of P1. Importantly, as shown in Fig. 2e, the delayed PL spectra of C1 remained unchanged at different excitation wavelengths in the crystal state under ambient conditions. The similar PL excitation spectra of P1 from 470 nm to 510 nm demonstrate that the RTP stemmed from the same triplet excited state but different energy levels (Fig. 2f). These results suggest that anti-Kasha’s rule was not responsible for the tunable afterglow color and that different molecular conformations were most likely responsible for the Ex-De RTP behaviors. It is speculated that various twisted conformations of C1 had different lowest-lying triplet states that were strictly fixed in a rigid environment, forming single emitters at different energy levels^29–32,39^; however, a highly ordered conformation is generally formed in the crystal state. This resulted in similar delayed PL spectra upon changing the excitation wavelength from 300 nm to 360 nm. Moreover, control monomers but-3-en-1-yltris(2-methoxyphenyl) phosphorane and but-3-en-1-yl(3-methoxyphenyl)bis(4-methoxyphenyl) phosphorane were designed and synthesized for copolymerization with acrylamide to prepare control polymers P6 and P7 (Supplementary Fig. 35). For P6, methoxyl groups are covalently linked on the ortho position of phenyl rings, which would restrict its rotary motions and thus twisted conformations are major ones in P6. In contrast, twisted and planar conformations were existed in P7, because its phenyl rings can be freely rotated. As expected, the phosphorescence peak of the P6 exhibited a small redshift (~ 5 nm), while P7 showed an evident bathochromic shift (~ 78 nm), upon changing the excitation wavelengths from 300 nm to 360 nm (Fig. 2e and Supplementary Fig. 35).

**Fig. 3:**
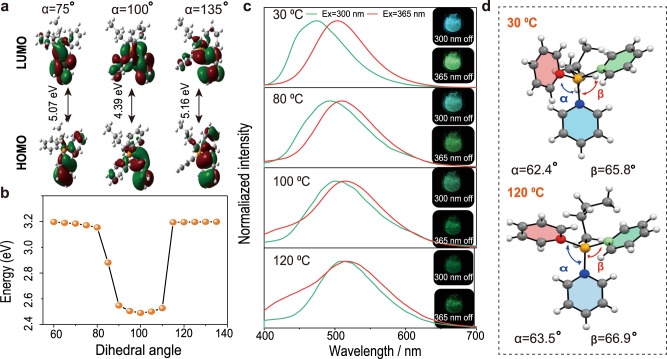
Theoretical calculation and thermal responsive RTP behavior. **a** Frontier molecular orbitals and excitation energies of the lowest excited triplet states of different C1 form molecules at TDA B3LYP/def2-SVP level of theory. **b** Relationship between the levels of the lowest-lying triplet states energy and changed dihedral angles of C1 by calculation. **c** Phosphorescence spectra of P1 upon heating from 30 °C to 120 °C, under 300 nm and 365 nm excitation. **d** Crystal structures of C1 obtained at 30 °C (CCDC 1977057) and 120 °C (CCDC 2224439).

To gain further insights into the mechanism of Ex-De persistent RTP, a relaxed scan based on the Tamm-Dancoff approximation (TDA) theory and B3LYP/def2-SVP level was carried out to calculate the phosphorescence spectrum of C1. As expected, the calculated energy levels of the lowest-lying triplet states varied as the dihedral angle changed from 75° to 135° (Fig. 3a and b). These results further confirmed that different molecular conformations in a rigid environment changed the PL spectra under different excitation wavelengths.

### Dynamic control of Ex-De RTP property through thermal energy

Considering the crucial role of the conformations of monomer for the Ex-De afterglow property, the dynamic manipulation of its response behavior is possible by controlling the molecular conformations in the polymer. The thermal energy can be used to drive molecular rotation in the solid state, as previously reported in the literature^40^. Fig. 3c shows that the delayed PL peak of P1 has an obvious red-shift from 474 to 506 nm, upon heating from 30 °C to 120 °C, under excitation at 300 nm; and the persistent luminescence color changes from a sky blue to a green. This is because molecular conformations varied along with the molecular rotations driven by the thermal stimulus. Also, the temperature strongly influenced the dynamic equilibrium among various excited states of phosphorescent rotors^39^. Upon heating, the molecular rotation of the monomer that crosses the thermal barriers of various excited states increases the population of the lower excited states. Thus, the phosphorescence peak of the heated P1 only showed a small red shift by varying the excitation wavelength from 300 to 365 nm, and the Ex-De RTP of P1 was turned “off”. After cooling to room temperature for 5 mins, the afterglow color of P1 was restored from green to sky blue under 300 nm excitation (Fig. 3c). Consequently, the Ex-De RTP behavior was switched “on” again. Therefore, the dynamic control of the Ex-De afterglow behavior of P1 was successfully demonstrated. Moreover, the phosphorescence peaks of P1 can be repeatedly varied for at least 10 times by changing the temperature (Supplementary Figs. 36 and 37), demonstrating its excellent reversibility.

To further reveal the relationship between conformation and phosphorescence properties, the delayed PL spectra and crystal structures of C1 were measured at room and high temperatures. When the temperature increased from 30 °C to 120 °C, the delayed PL spectra of C1 showed a red shift about 10 nm under 300 nm excitation (Supplementary Fig. 38). In addition, the dihedral angles of phenyl rings of C1 changed from 62.4° and 65.8° to 63.5° and 66.9°, respectively, when the temperature raised (Fig. 3d). This result indicate that the phosphorescence property is directly associated with the conformation of C1. Furthermore, the theoretical calculation was carried out to evaluate the energy levels of C1 at different temperatures. As expected, its lowest-lying triplet states changed with conformation varied (Supplementary Figs. 39 and 40), which can be well accordant with the experimental results.

### Demonstration on the universality of the design principle

**Fig. 4:**
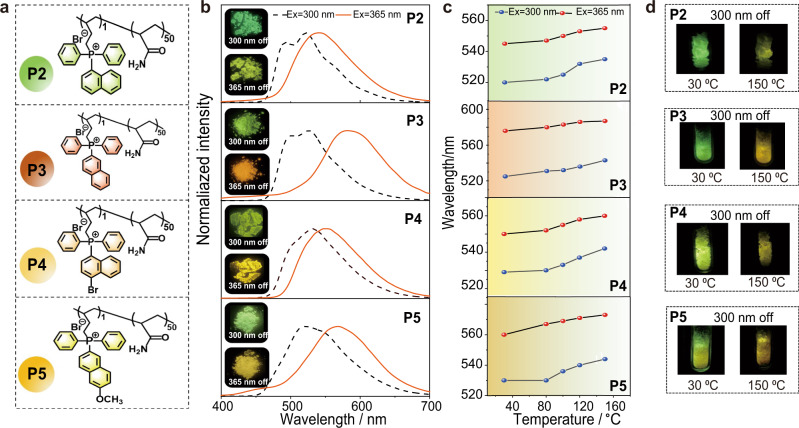
Chemical structures of P2-P5 and their RTP properties. **a** Chemical structure of P2-P5. **b** Ex-De phosphorescence spectra of P2-P5, and their photographs under 300/365 nm excitation. **c** Phosphorescence spectra of P2-P5 upon heating from 30 °C to 120 °C. **d** Photographs of P2-P5 upon heating from 30 °C to 150 °C under 300 nm excitation.

To demonstrate the universality of our strategy, the other four phosphorescent rotors of but-3-en-1-yl(naphthalen-1-yl)diphenylphosphonium bromide (M2), butyl(naphthalen-2-yl)diphenylphosphonium bromide (M3), (4-bromonaphthalen-1-yl)(but-3-en-1-yl)diphenylphosphonium bromide (M4), and butyl(6-methoxynaphthalen-2-yl)diphenylphosphonium bromide (M5) were also incorporated into polyacrylamide, namely P2-P5, with copolymerization ratio of 1/50 (Fig. 4a). The photophysical properties of monomers M2-M5 are given in Supplementary Fig 41. As expected, these polymers showed obvious Ex-De afterglow behaviors. As displayed in Fig. 4b, the delayed PL maxima of P2-P5 could be changed from 500 nm to 548 nm, 525 nm to 588 nm, 530 nm to 560 nm, 520 nm to 570 nm, respectively, under 300 and 365 nm excitation (Supplementary Figs. 42–50, Supplementary Tab 3), accompanying with afterglow color transitions from green (CIE, 0.26, 0.56) to yellow- green (CIE, 0.32, 0.58), green (CIE, 0.28, 0.58) to orange (CIE, 0.49, 0.47), green-yellow (CIE, 0.38, 0.59) to orange (CIE, 0.42, 0.51), green-yellow (CIE, 0.33, 0.55) to yellow-orange (CIE, 0.43, 0.53) (Supplementary Fig. 51).

Importantly, the Ex-De RTP behaviors of these polymers can be controlled by thermal energy as well. For example, the delayed PL peak of P3 has an obvious red-shift from 525 nm to 547 nm upon heating from 30 °C to 150 °C, under excitation at 300 nm (Fig. 4c and d). Similarly, a small bathochromic shift from 588 to 593 nm was observed upon heating 30 °C to 150 °C, under 365 nm excitation. Therefore, for P3, its afterglow color changed from green to orange upon varying excitation wavelength at room temperature, while the persistent luminescence varied from yellow to orange was observed by changing excitation energies at high temperature (Fig. 4c and d). The changes in response behavior of P3 is different from P1, which might be because the rotation ability of naphthyl group is different from phenyl group. For P2-P5, they exhibited similar changes in Ex-De RTP behavior upon thermal treatment, demonstrating the controllable response behaviors for these materials (Fig. 4c and d).

### Multi-level anti-counterfeiting applications

**Fig. 5:**
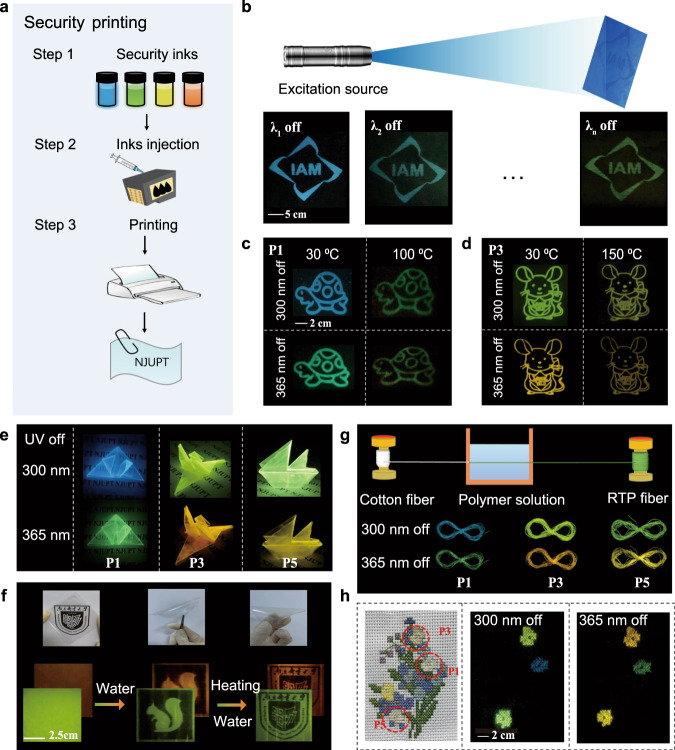
Transparent films and cotton fibers with Ex-De afterglow behavior. **a** Schematic illustration the preparation of transparent films for water-jet printing. **b** Photographs of different printed images on a A4 filter paper by using P1 as the ink before and after removing UV irradiation. **c** Photographs of printed patterns on filter paper by using P1 and P3 as inks, upon heating from 30 °C to 100 °C. **d** Photographs of printed patterns on filter paper by using P3 as inks, upon heating from 30 °C to 150 °C. **e** Photographs of different transparent 3D objects after removing 300/365 nm irradiation. **f** Photographs of P3-doped transparent films for rewritable water-jet printing. **g** Schematic illustration the preparation of persistent RTP fibers. **h** Photographs of embroidered patterns under the daylight and after removing 300 nm and 365 nm irradiation.

The fantastic dynamic manipulation of the Ex-De persistent RTP behavior is appealing for multi-level anti-counterfeiting applications. For use in practical applications, organic persistent RTP materials must have good processability. Supplementary Fig. 53 shows powder X-ray diffraction (PXRD) patterns of P1-P5 which contain a broad diffraction peak with a very weak intensity, indicating their amorphous nature. These results suggest that these unique Ex-De persistent RTP colors can be achieved in the amorphous state, giving the material great potential for processing and practical applications. It is worth noting that these polymers are excellent candidates for practical applications due to their large-area processability and flexibility. For example, P1 and P3 can be used to dissolve in the aqueous solution to prepare security inks, coupled with a commercial ink-jet printer (Fig. 5a), large-area security printing of high-resolution patterns was successfully achieved. As show in Fig. 5b, multicolor afterglow labels could be clearly seen after removing the UV sources (Supplementary Figs. 54 and 55). Subsequently, upon heating the printed patterns, we can clearly see changes in afterglow colors under excitation at 300 nm and 365 nm (Fig. 5c and d). These results demonstrated the potential importance of these polymers in the produce of anticounterfeiting tags, since the security of the created pattern is remarkably promoted because of the dynamic manipulation of Ex-De afterglow properties.

Next, we fabricated large-area transparent poly(vinylalcohol) (PVA) films containing these persistent RTP polymers^41,42^. As shown in Fig. 5e, large-area transparent 3D objects were fabricated by folding and bending the prepared PVA films containing P1, P3 or P5. After ceasing the UV light excitation, visible multicolor persistent RTP from P1, P3 or P5 doped PVA films were observed under the ambient conditions. Besides, the transparent films could be used for rewritable printing of Ex-De persistent RTP patterns by using aqueous solution as the ink. Figure 5f showed that various delicate images can be printed on the P3-doped transparent film by using a daily used inkjet printer. By heating the film with a blower for about 5 mins, the recorded patterns can be erased. Then, the other information or images can be recorded again by using water as the ink. In addition, functional fibers with tunable RTP colors were prepared to produce smart-responsive textiles (Fig. 5g and h). The prepared fibers exhibit the similar Ex-De persistent RTP properties with their corresponding polymers. We then used these fibers to produce embroidered patterns, which exhibited multicolor afterglow RTP under different excitation wavelengths, demonstrating their potential applications in flexible and wearable electronics.

## Discussion

In summary, we present an effective strategy to achieve controllable Ex-De persistent luminescence in a series of amorphous polymers. Important insights into the relationship between conformation of monomer and Ex-De persistent RTP behaviors have been obtained through both experimental results and theoretical calculations. Thus, the dynamic manipulation of Ex-De persistent RTP was unprecedentedly achieved by thermal energy-driven molecular rotations. On the basis of this unique characteristic, high-level anti-counterfeiting labels can be produced through ink-jet printing technique, and their Ex-De afterglow color changed with the temperature varied. In addition, large-area transparent films and fibers with Ex-De afterglow emissions were prepared for security applications. It is believed that this finding would break the traditional understanding of the manipulation of response behaviors of RTP materials, while offering the possibility for the dynamic control of response behaviors through external stimuli.

## Methods

### Measurements

^1^H NMR (400 MHz) and ^13^C NMR (100 MHz) spectra were recorded on a Bruker ACF400 spectrometer at 298 K using deuterated solvents (CDCl_3_ or DMSO-*d*_*6*_). The UV-visible absorption spectra were measured by Shimadzu UV-2600 UV-vis spectrophotometer. The steady-state fluorescence and phosphorescence spectra were measured using Hitachi F-4700. The lifetimes were obtained on an Edinburgh FLS980 fluorescence spectrophotometer equipped with a Xenon arc lamp (Xe900) and a microsecond flash-lamp (uF900). The powder X-ray diffraction patterns were collected by D8 Advanced (Bruker) using Cu-Kα radiation. Aqueous gel permeation chromatography (GPC) was performed on a Series Samples used 0.05 mol/L NaNO_3_/H_2_O as mobile phase at 1.0 mL min^–1^ flow rate. PEG was used as the calibration standard. The crystalline samples were obtained from slow evaporation of a mixture of hexane/dichloromethane (1:1, v/v). ^13^C NMR (100 MHz) spectra were measured recorded on a Bruker ACF400 spectrometer at 298 K using deuterated solvents.

### Preparation of transparent films

Firstly, PVA-1799 (2.0 g) and P1 (400 mg) were dissolved in deionized water (20 mL). Then, the mixture was stirred at 108 °C for 6 h. The films were produced with drop-casting approach using the mixed solution on a glass plate. After drying at 45 °C for 12 h, and exfoliation, the lager-area transparent films were obtained. Lastly, various large-area transparent objects using the P1 film were prepared by rolling, folding, bending, and extending approaches under ambient conditions. P2, P3, P4 and P5 films were obtained in a similar way.

### Preparation of RTP fibers

First, P1 (1.0 g) was dissolved in 10 mL of deionized water, followed by the sonication for 10 mins under ambient conditions. Subsequently, the solution was poured into a tank, and the cotton fibers were soaked in the solution for 24 h and then heated at 65 °C for 2 h to obtain RTP fiber. P3 and P5 fibers were prepared in a same way.

### Computational details

The theoretical calculations were performed via the Gaussian 16 suite of program (Revision A.03). The structures of the studied systems were extracted from the cif files at different temperature. The systems at the triplet excited state were calculated at the TDA B3LYP/def2-SVP level of theory. The molecular orbital levels of studied complexes were investigated via theoretical calculations, including the highest occupied molecular orbital (HOMO) and the lowest unoccupied molecular orbital (LUMO). The Visual Molecular Dynamics (VMD) program was used to plot the color-filled iso-surface graphs to visualize the molecular orbitals.

## Supplementary information

Supplementary Information

## Data Availability

The datasets generated during and/or analyzed during the current study are available from the corresponding author on request. Source data are provided with this paper.

## Source data

Source Data
