# Supplementary material for: Conformation-dependent dynamic organic phosphorescence through thermal energy driven molecular rotations
Source: Nat Commun. 2023 Feb 6;14:627. doi: 10.1038/s41467-023-35930-5 (PMC9902600; doi:10.1038/s41467-023-35930-5)
Supplement: Supplementary file 1 — Supplementary Information [file 41467_2023_35930_MOESM1_ESM.pdf]

Supplementary Information

Experimental Procedures

Materials. Unless otherwise stated, all starting materials and reagents were purchased from commercial suppliers and used without further purification. All solvents were purified before use. The solvents were carefully dried and distilled from appropriate drying agents prior to use.

Synthesis and characterization

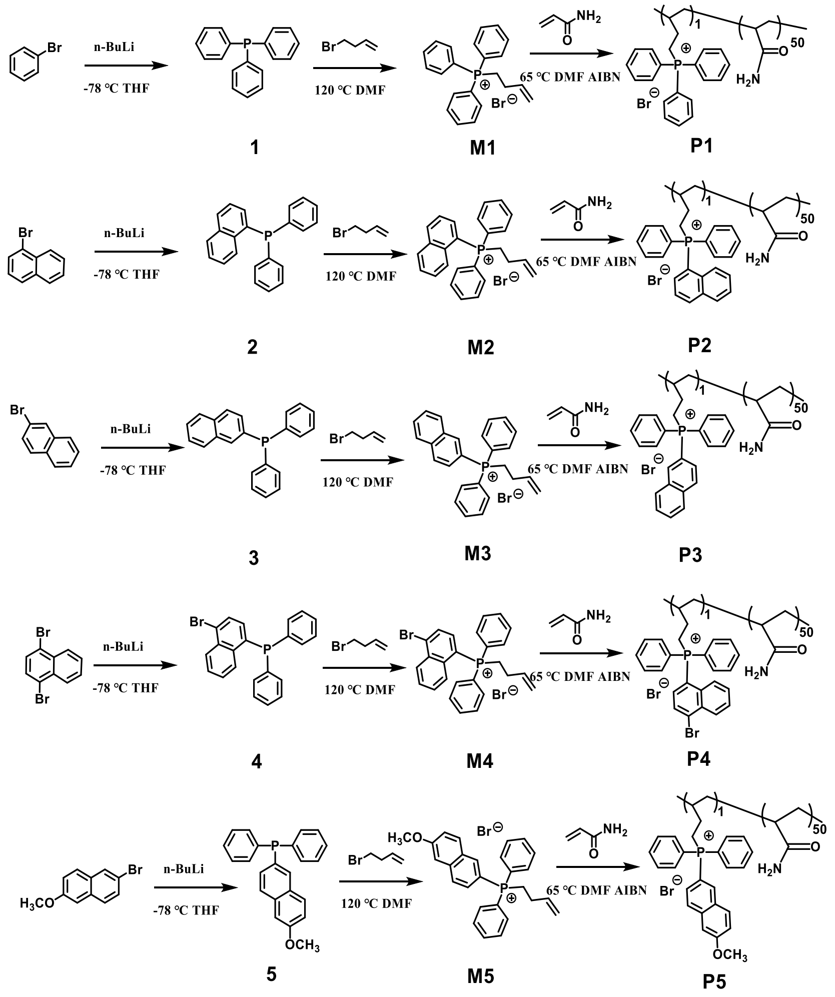

Supplementary Fig. 1 Synthesis. Synthetic route of M1-M5 and P1-P5.

Synthesis of M1:

The 1.6 M hexane solution of *n*-BuLi (24.0 mL, 38.46 mmol) was added dropwise to an agitating superdry THF solution (40 mL) of bromobenzene (5.0 g, 32.05 mmol) at -78 °C under an inert atmosphere of nitrogen. After stirring for 1 h, chlorodiphenylphosphine (7.0 g, 32.05 mmol) was added into the mixture. The reaction was stirred at 25 °C for 12 h. 1 was purified through column chromatography (petroleum ether), yield 85%. In nitrogen atmosphere, compound 1 (2 g, 7.62 mmol) was added into the mixture of 4-Bromo-1-butene (1.5 g, 9.15 mmol).and DMF (50 mL). The reaction was stirred at 120 °C for 24 h. M1 was purified through column chroma-tography (methanol : dichloromethane = 50 : 1), yield 90%. ^{1}H NMR (400 MHz, $CDCl_3$, δ): 7.92-6.64 (m, 15H), 5.99 (m, 1H), 5.06 (m, 1H), 5.00 (d, 1H), 3.99-3.84 (m, 2H), 2.52-2.37 (m, 2H). ^{13}C NMR (100 MHz, $CDCl_3$, δ): 135.17, 135.14, 134.97, 134.82, 133.78, 133.68, 130.63, 130.51, 118.53, 117.67, 117.46, 26.66, 26.63, 22.62, 22.13. HRMS: m/z = 317.3964 [M − Br].

Synthesis of M2:

The 1.6 M hexane solution of *n*-BuLi (18.1 mL, 28.98 mmol) was added dropwise to an agitating superdry THF solution (40 mL) of 1-bromonaphthalene (5 g, 24.15 mmol) at -78 °C under an inert atmosphere of nitrogen. After stirring for 1 h, chlorodiphenylphosphine (5.3 g, 24.15 mmol) was added into the mixture. The reaction was stirred at 25 °C for 12 h. 2 was purified through column chromatography (petroleum ether), yield 80%. In nitrogen atmosphere, 2 (2 g, 6.41 mmol) was added into the mixture of 4-bromo-1-butene (1.0 g, 7.69 mmol) and DMF (20 mL). The reaction was stirred at 120 °C for 24 h. The M2 was purified through column chromatography (methanol : dichloromethane = 50 : 1), yield 88%. ^{1}H NMR (400 MHz, $CDCl_3$, δ): 8.43-8.28 (m, 1H), 8.20-8.03 (m, 2H), 7.95-7.60 (m, 13H), 7.58-7.46 (m, 1H), 6.03-5.88 (m, 1H), 4.93 (s, 2H), 4.14-3.90 (m, 2H), 2.51-2.27 (m, 2H). ^{13}C NMR (100 MHz, $CDCl_3$, δ): 138.10, 136.86, 135.49, 135.16, 133.99, 131.98, 131.00, 129.08, 127.71, 125.39, 119.54, 113.60, 26.48, 21.40. HRMS: m/z = 367.4524 [M − Br].

Synthesis of M3:

The 1.6 M hexane solution of *n*-BuLi (18.1 mL, 28.98 mmol) was added dropwise to an agitating superdry THF solution (40 mL) of 2-bromonaphthalene (5 g, 24.15 mmol) at -78 °C under an inert atmosphere of nitrogen. After stirring for 1 h, chlorodiphenylphosphine (5.3 g, 24.15 mmol) was added into the mixture. The reaction was stirred at 25 °C for 12 h. 3 was purified through column chromatography (petroleum ether), yield 86%. In nitrogen atmosphere, 3 (2 g, 6.41 mmol) was added into the mixture of 4-bromo-1-butene (1.0 g, 7.69 mmol) and DMF (20 mL). The reaction was stirred at 120 °C for 24 h. The M3 was purified through column chromatography (methanol : dichloromethane = 50 : 1), yield 80%. ^{1}H NMR (400 MHz, $CDCl_3$, δ): 8.79 (d, 1H), 8.21 (d, 1H), 8.12 (m, 1H), 7.93 (m, 5H), 7.83 (m, 2H), 7.78-7.65 (m,

7H), 6.07 (m, 1H), 5.14-4.98 (m, 2H), 4.18-4.01 (m, 2H), 2.61-2.43 (m, 2H). ^{13}C NMR (100 MHz, $CDCl_3$, δ): 137.39, 137.29, 135.53, 135.16, 135.04, 134.90, 133.77, 133.68, 132.81, 132.67, 130.63, 130.51, 129.75, 128.36, 128.00 126.11, 126.01, 118.76, 117.90, 117.44, 114.88, 114.02, 26.75, 26.71, 22.74, 22.25. HRMS: m/z = 367.4524 [M − Br].

Synthesis of M4:

The 1.6 M hexane solution of *n*-BuLi (13.1 mL, 20.98 mmol) was added dropwise to an agitating superdry THF solution (40 mL) of 1,4-dibromonaphthalene (5 g, 17.48 mmol) at -78 °C under an inert atmosphere of nitrogen. After stirring for 1 h, chlorodiphenylphosphine (4.6 g, 20.98 mmol) was added into the mixture. The reaction was stirred at 25 °C for 12 h. 4 was purified through column chromatography (petroleum ether), yield 81%. In nitrogen atmosphere, 4 (2 g, 5.11 mmol) was added into the mixture of 4-bromo-1-butene (2.3 g, 10.23 mmol) and DMF (20 mL). The reaction was stirred at 120 °C for 24 h. The M4 was purified through column chromatography (methanol : dichloromethane = 50 : 1), yield 65%. ^{1}H NMR (400 MHz, DMSO-d_6, δ): 8.77-8.54 (m, 1H), 8.63 -8.49 (m, 1H), 8.42-8.24 (m, 1H), 8.26-8.11 (m, 1H), 7.91-7.73 (m, 13H), 6.14 -5.90 (m, 1H), 5.27-5.03 (m, 2H), 3.93-3.55 (m, 2H), 2.40-2.25 (m, 2H). ^{13}C NMR (100 MHz, DMSO-d_6, δ): 137.52, 133.41, 130.60, 119.13, 118.88, 23.48, 22.01, 19.94, 13.20. HRMS: m/z = 446.3510 [M − Br].

Synthesis of M5:

The 1.6 M hexane solution of *n*-BuLi (15.8 mL, 25.32 mmol) was added dropwise to an agitating superdry THF solution (40 mL) of 2-bromo-6-methoxynaphthalene (5.0 g, 21.09 mmol) at -78 °C under an inert atmosphere of nitrogen. After stirring for 1 h, chlorodiphenylphosphine (4.6 g, 21.09 mmol) was added into the mixture. The reaction was stirred at 25 °C for 12 h. 5 was purified through column chromatography (petroleum ether), yield 77%. In nitrogen atmosphere, 5 (2 g, 5.85 mmol) was added into the mixture of 4-bromo-1-butene (0.95 g, 7.01 mmol) and DMF (20 mL). The reaction was stirred at 120 °C for 24 h. The M5 was purified through column chromatography (methanol : dichloromethane = 50 : 1), yield 88%. ^{1}H NMR (400 MHz, $CDCl_3$, δ): 8.67-8.49 (m, 1H), 8.04 (d, 2H), 7.91-7.64 (m, 10H), 7.61-7.53 (m, 1H), 7.21 (d, 1H), 6.21-5.82 (m, 1H), 5.20-4.75 (m, 2H), 3.95 (s, 5H), 2.61-2.34 (m, 2H), 1.98-1.85 (m, 1H). ^{13}C NMR (100 MHz, $CDCl_3$, δ): 161.11, 137.82, 137.00, 134.39, 131.28, 130.29, 129.27, 128.26, 126.78, 121.47, 119.42, 118.43, 111.65, 110.42, 106.58, 56.65, 26.70, 27.01, 23.17. HRMS: m/z = 397.4723 [M − Br].

Synthesis of P1-P5:

Compound M1-M5 were copolymerized with acrylamide at the molar ratio of 800:1, 400:1, 200:1, 100:1, 50:1, 10:1 in order to prepare amorphous polymers (P1-P5). In view of the similarity of the synthesis method, P1 (1/50) was used as an example for demonstration. The polymer was prepared by copolymerization of the compound M1 (23.7 mg, 0.06 mmol, 1 eq) and acrylamide (212.2 mg, 3.0 mmol, 50 eq) by a radial polymerization with 2,2'-azobis(2-methylpropionitrile) (AIBN) (4 mg) as radical initiator at 65°C under an argon atmosphere in DMF for 12 h. The resulting mixture was added into methanol to precipitate polymeric materials. Precipitation was repeatedly washed with methanol to give purified polymers.

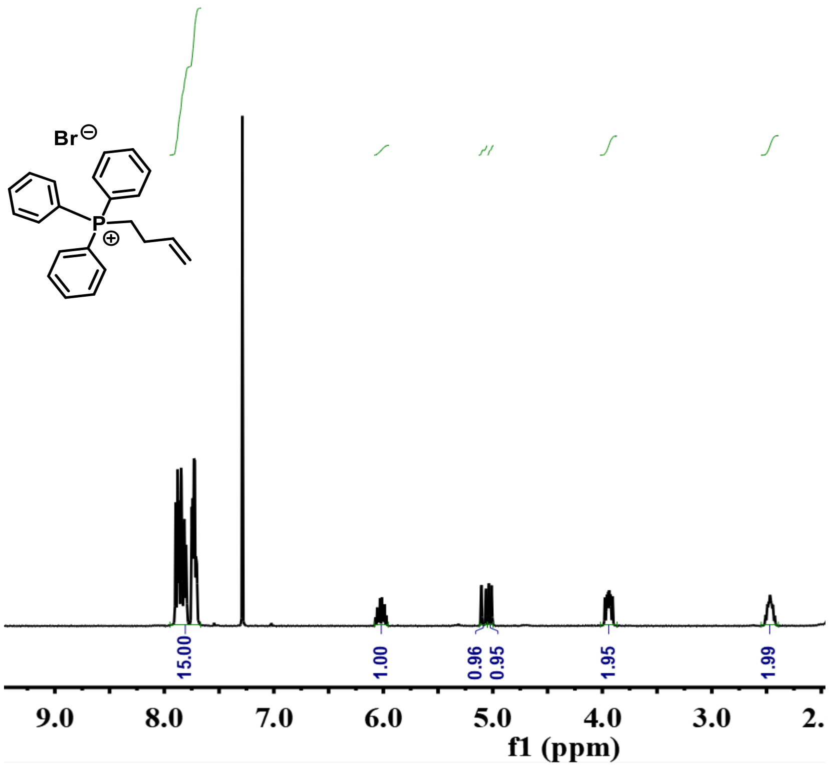

Supplementary Fig. 2 ^{1}H NMR spectrum of M1 in $CDCl_3$.

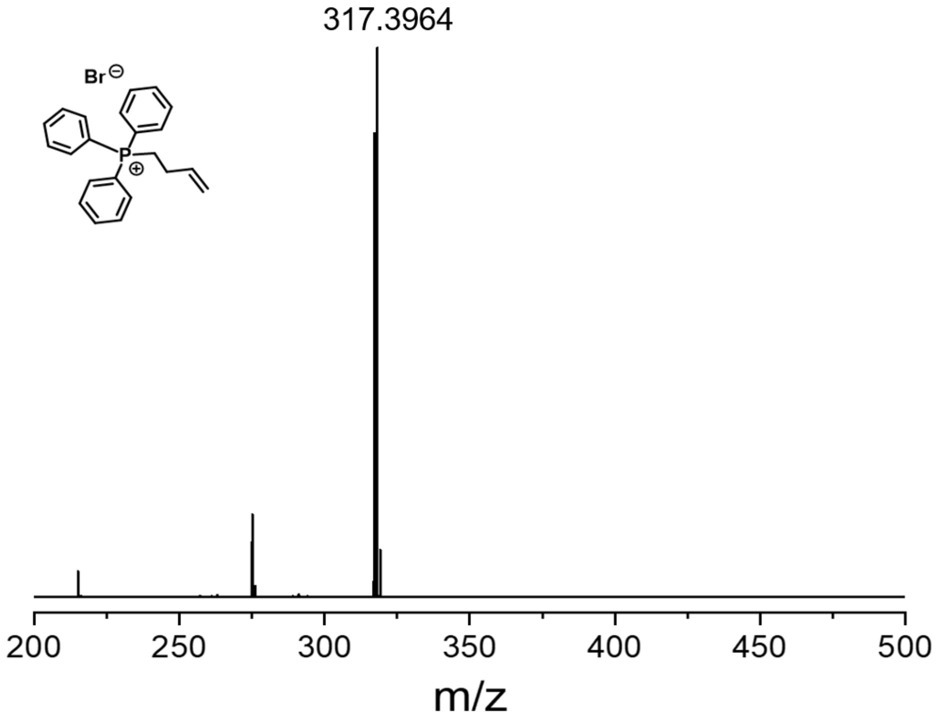

Supplementary Fig. 3 HRMS spectrum of M1.

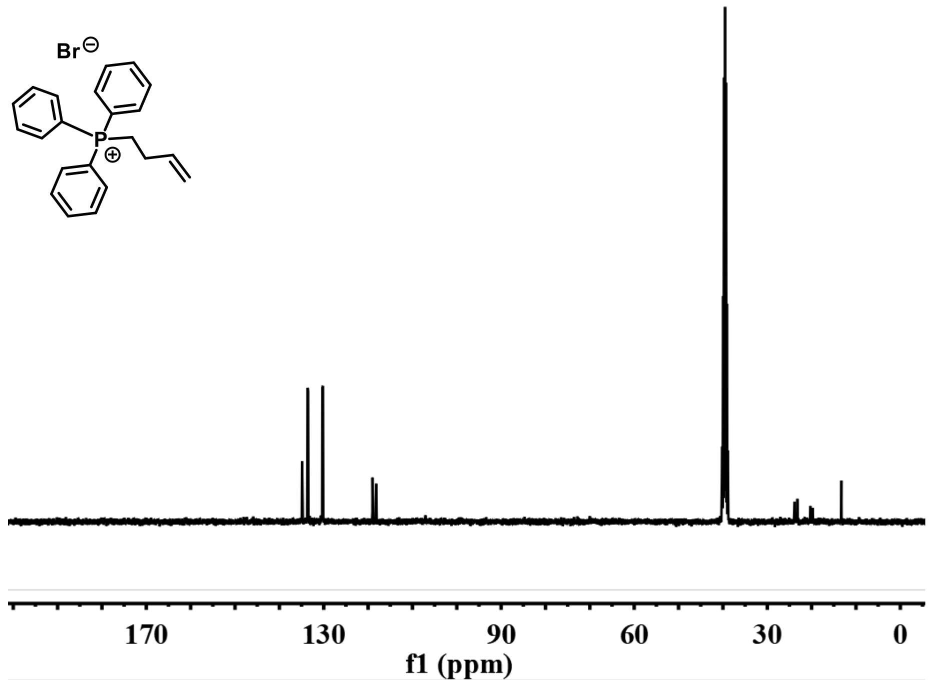

Supplementary Fig. 4 ^{13}C NMR spectrum of M1 in DMSO-d_6.

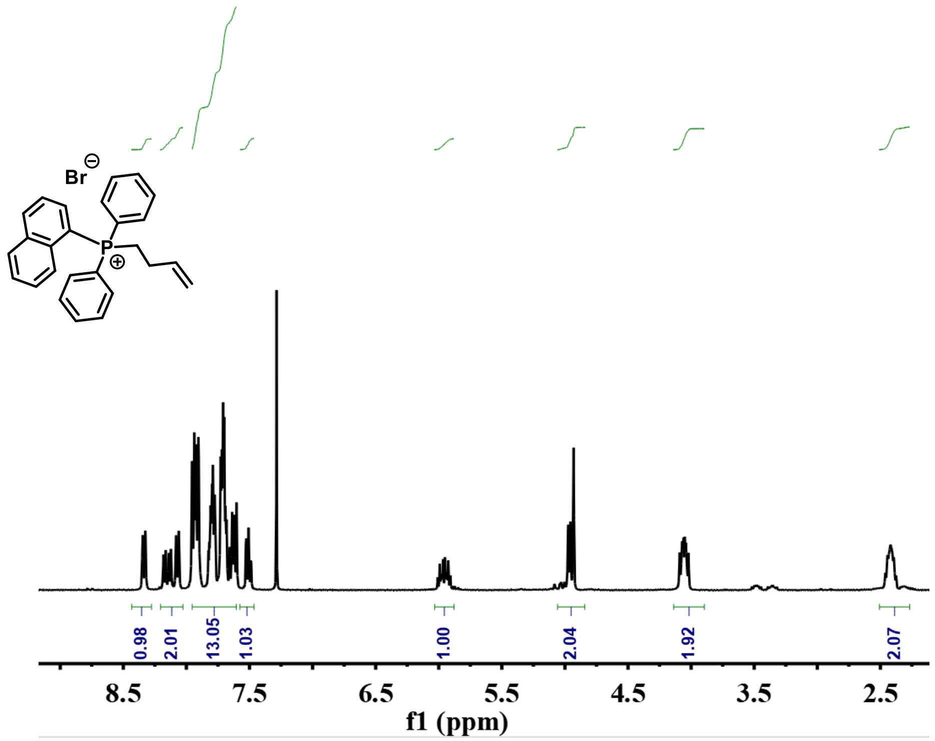

Supplementary Fig. 5 ^{1}H NMR spectrum of M2 in CDCl$_3$.

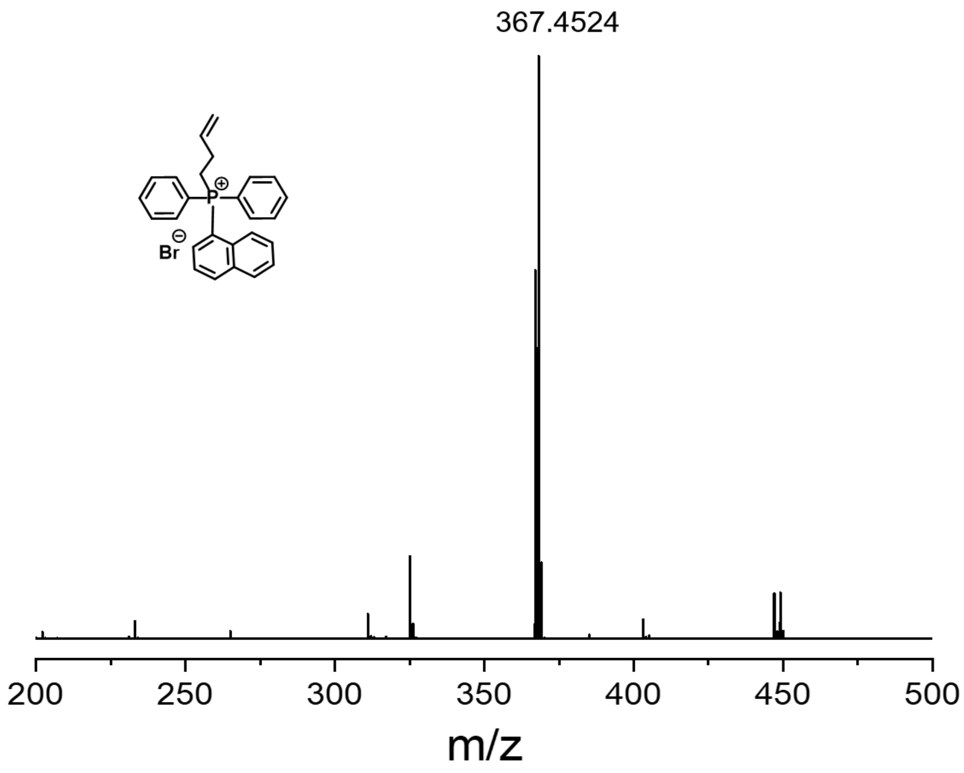

Supplementary Fig. 6 HRMS spectrum of M2.

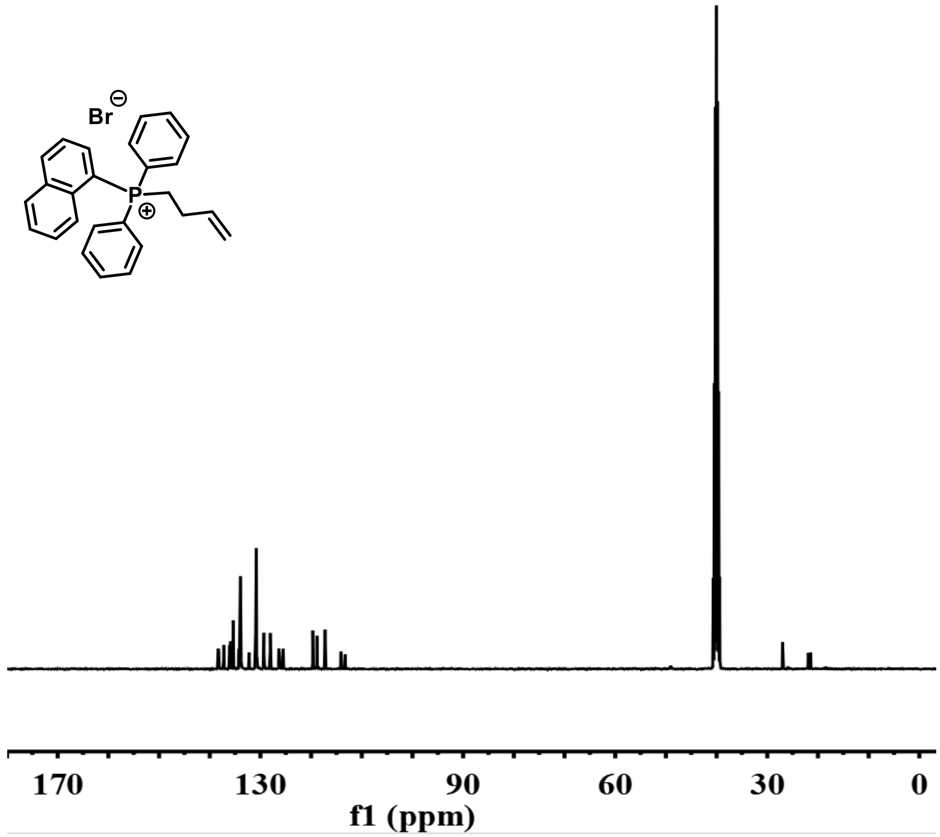

Supplementary Fig. 7 ^{13}C NMR spectrum of M2 in DMSO-d_6.

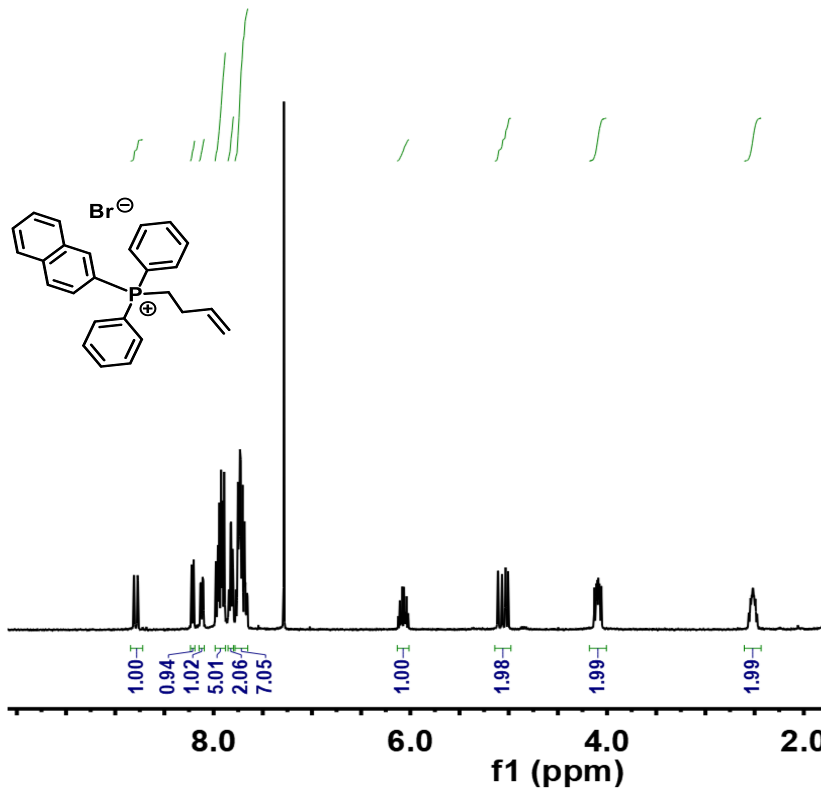

Supplementary Fig. 8 ^{1}H NMR spectrum of M3 in $CDCl_3$.

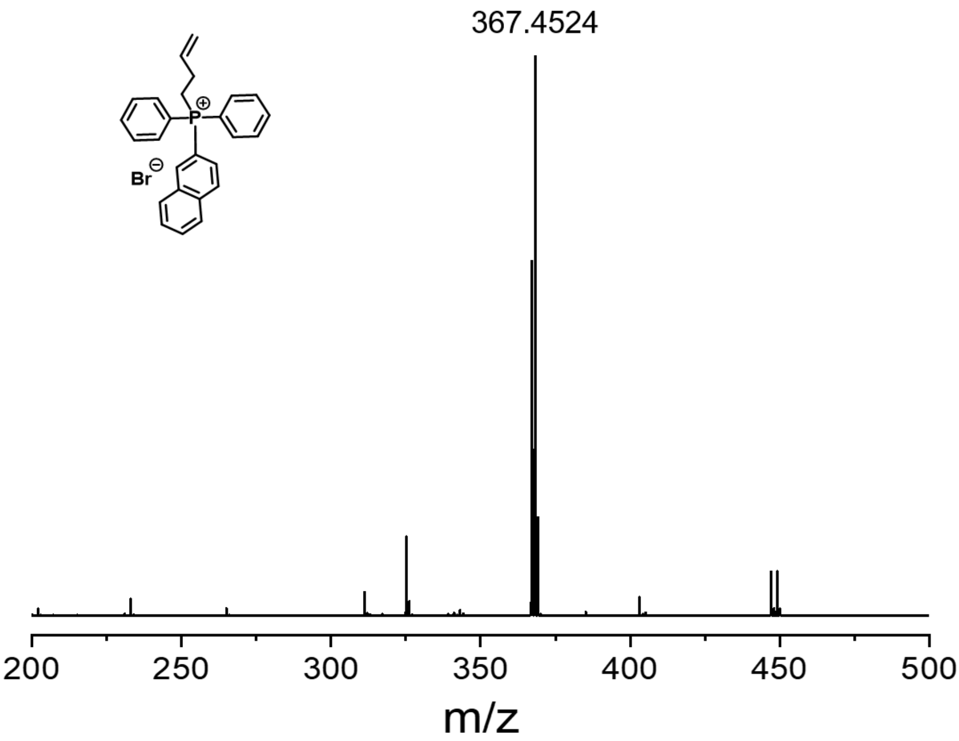

Supplementary Fig. 9 HRMS spectrum of M3.

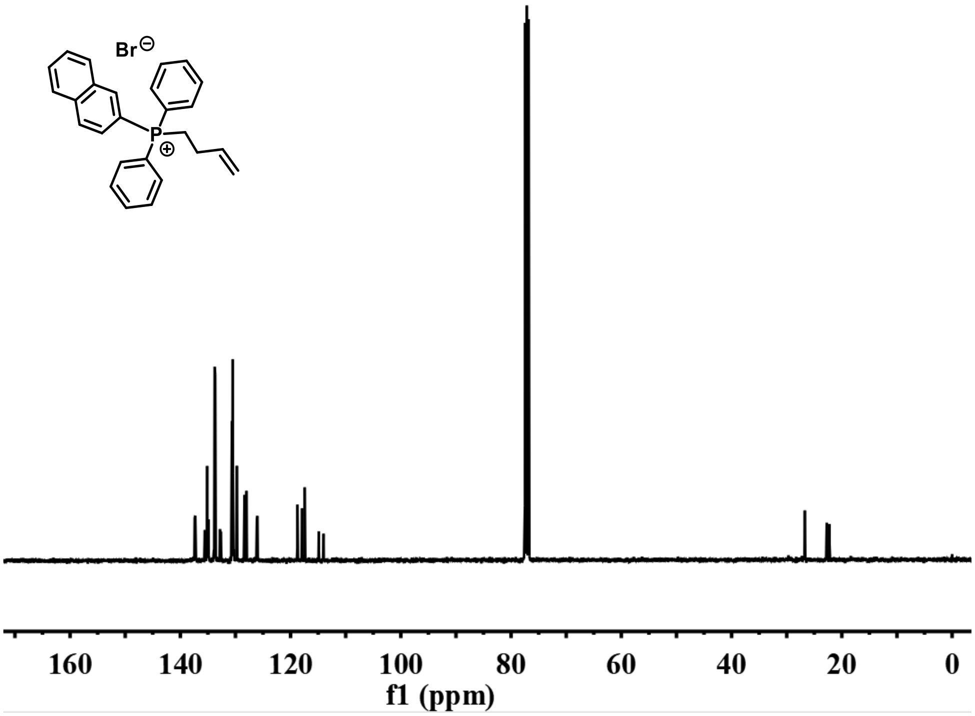

Supplementary Fig. 10 ^{13}C NMR spectrum of M3 in $CDCl_3$.

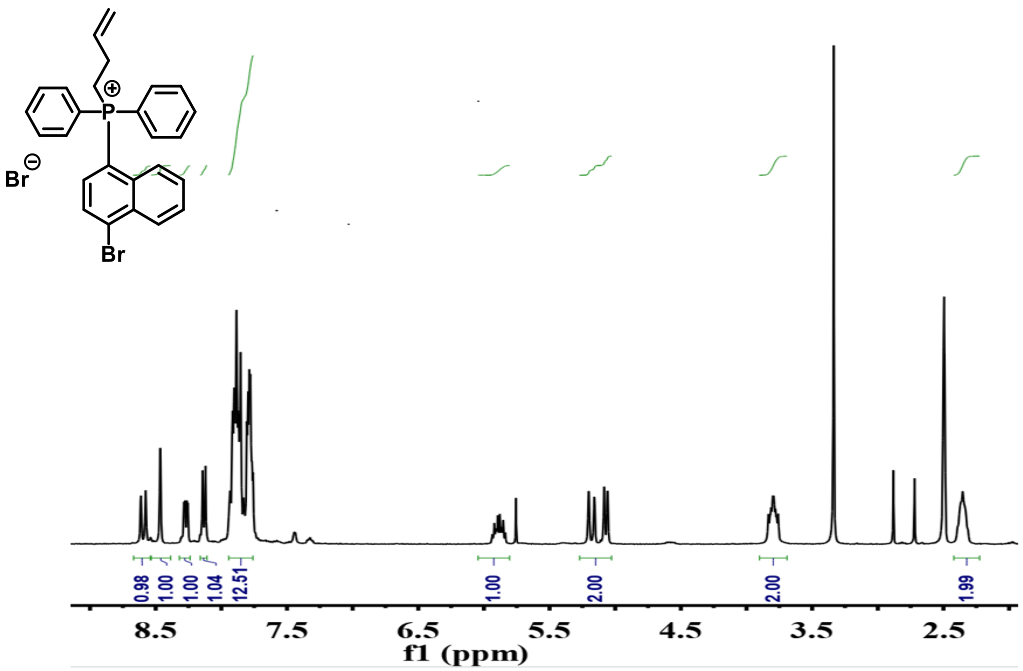

Supplementary Fig. 11 ^{1}H NMR spectrum of M4 in DMSO-d_6.

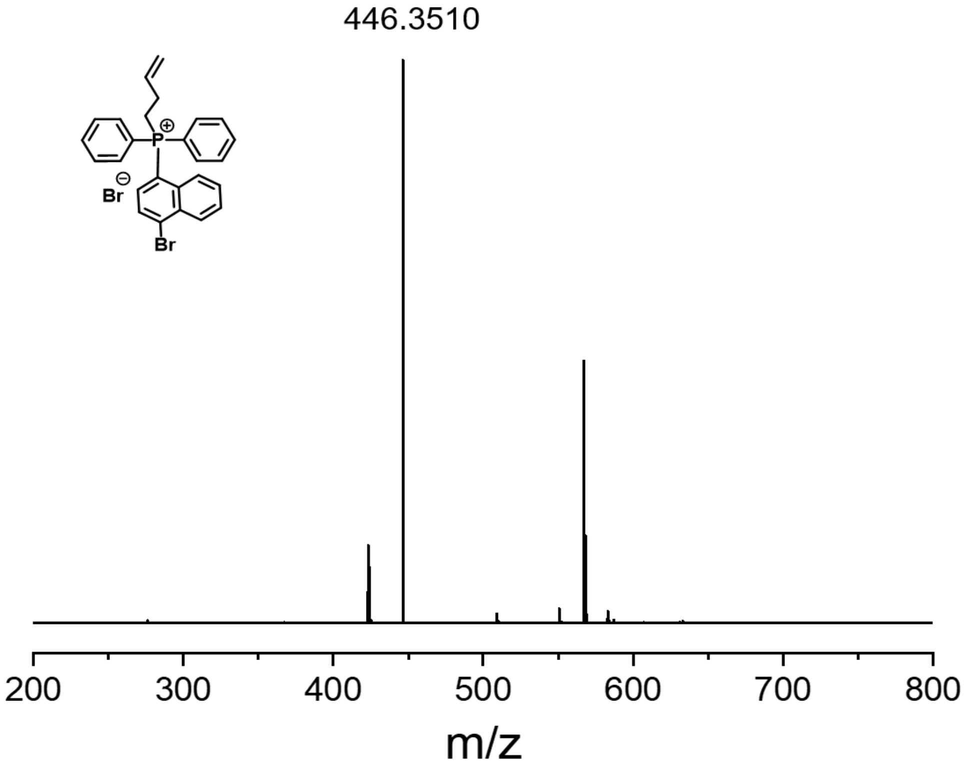

Supplementary Fig. 12 HRMS spectrum of M4.

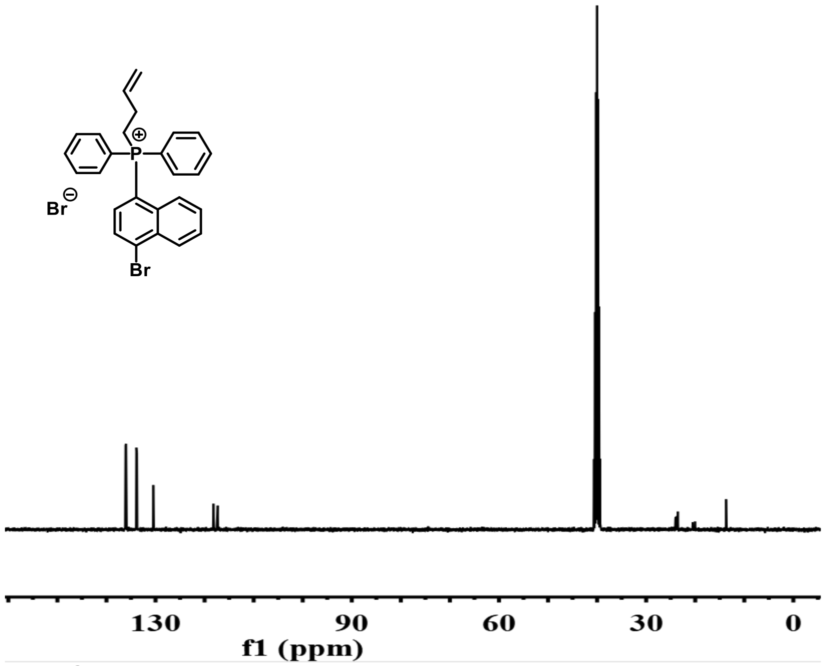

Supplementary Fig. 13 ^{13}C NMR spectrum of M4 in DMSO-d_6.

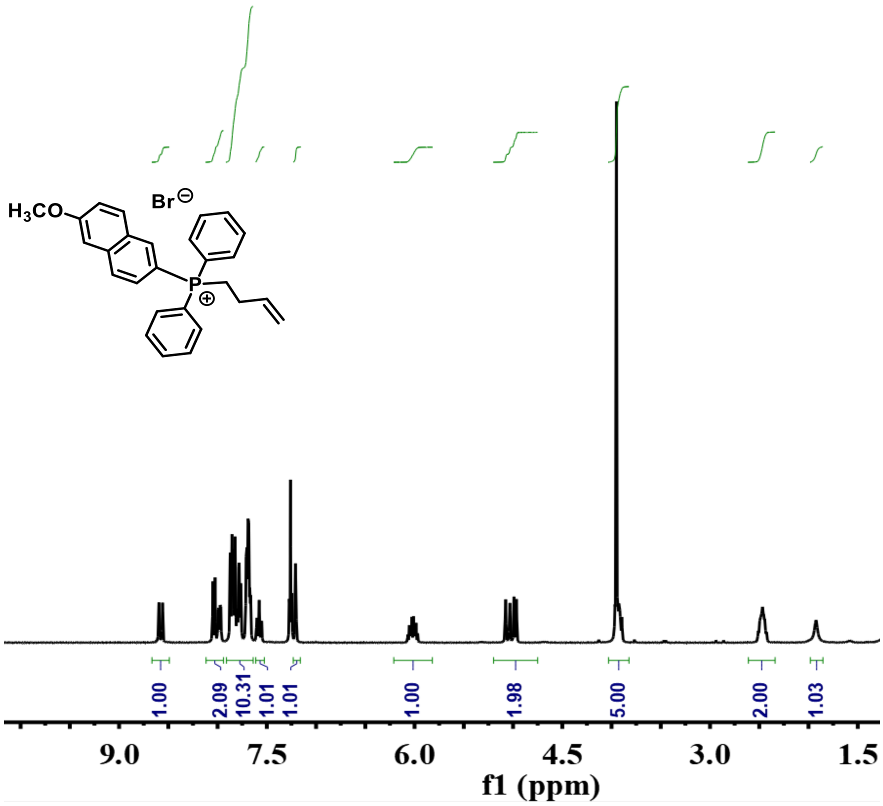

Supplementary Fig. 14 ^{1}H NMR spectrum of M5 in $CDCl_3$.

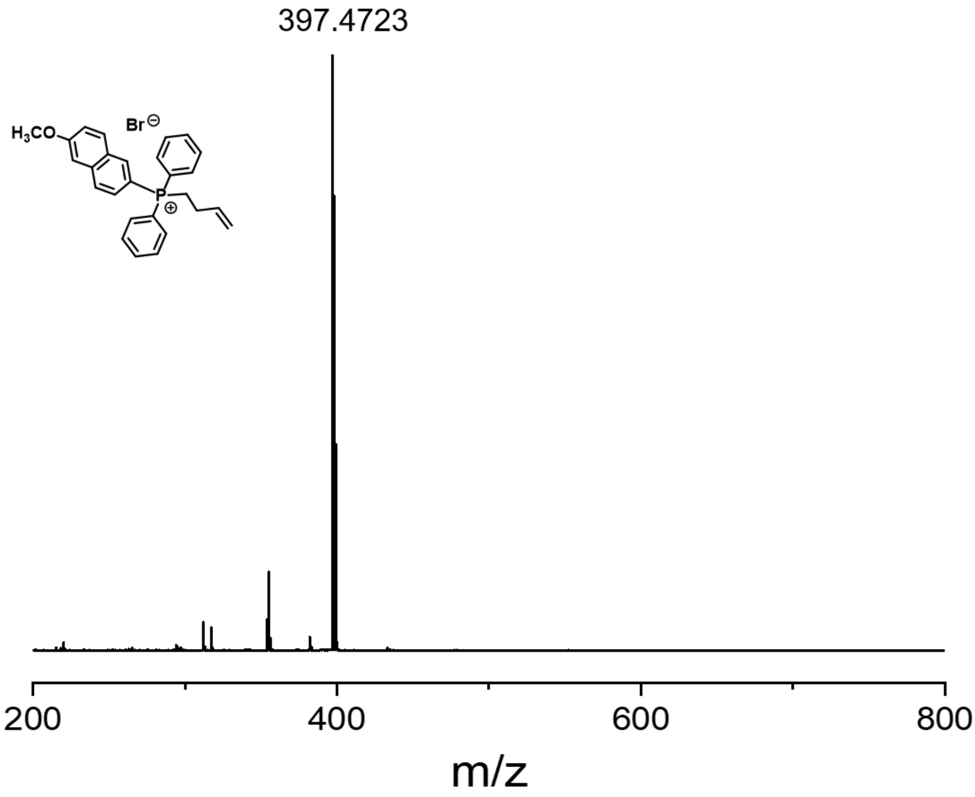

Supplementary Fig. 15 HRMS spectrum of M5.

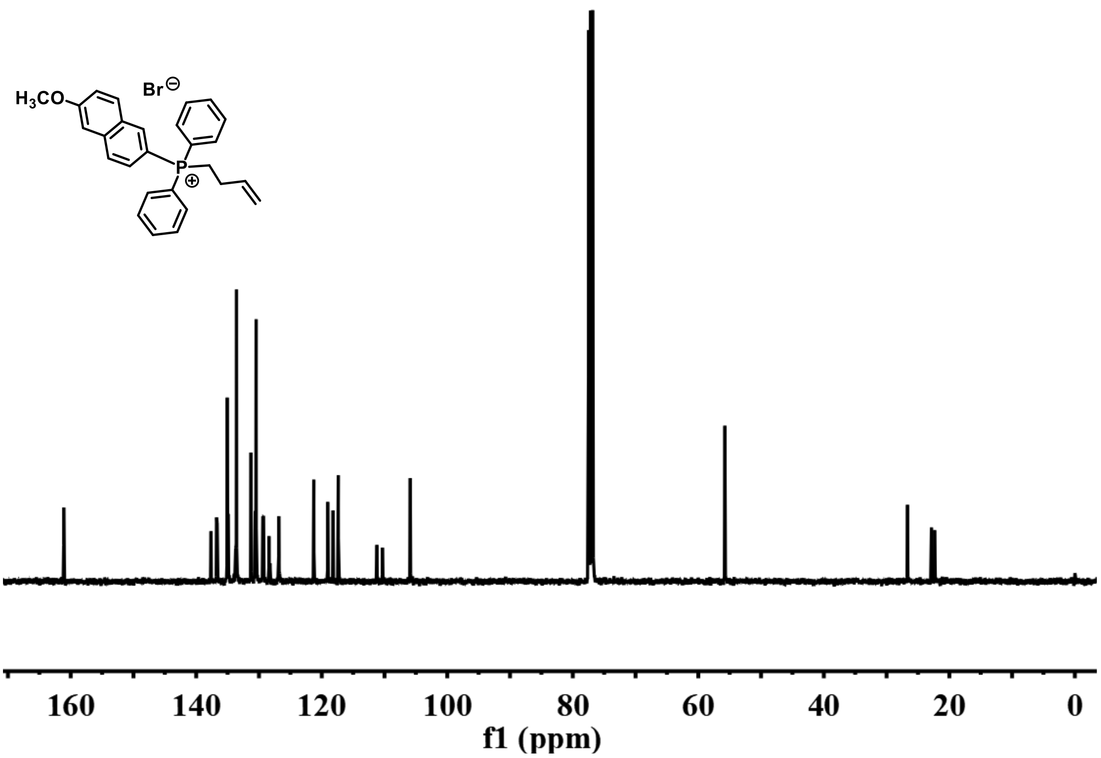

Supplementary Fig. 16 ^{13}C NMR spectrum of M5 in $CDCl_3$.

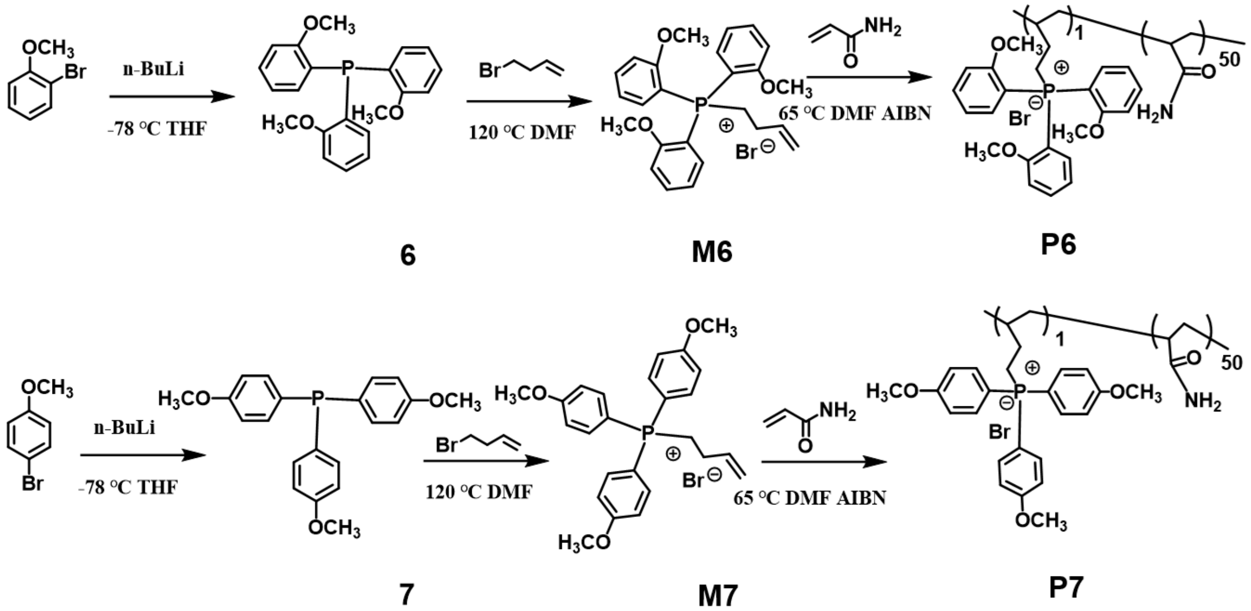

Supplementary Fig. 17 Synthesis. Synthetic route of P6-P7**.**

Synthesis of M6:

The 1.6 M hexane solution of *n*-BuLi (20.00 mL, 32.08 mmol) was added dropwise to an agitating superdry THF solution (40 mL) of 2-bromoanisole (5 g, 26.73 mmol) at -78 °C under an inert atmosphere of nitrogen. After stirring for 1 h, chlorodiphenylphosphine (5.8 g, 26.73 mmol) was added into the

mixture. The reaction was stirred at 25 °C for 12 h. 6 was purified through column chromatography (petroleum ether), yield 82%. In nitrogen atmosphere, 6 (2 g, 5.68 mmol) was added into the mixture of 4-bromo-1-butene (1.53 g, 11.4 mmol) and DMF (20 mL). The reaction was stirred at 120 °C for 24 h. The M6 was purified through column chromatography (methanol : dichloromethane = 50 : 1), yield 83%. ^{1}H NMR (400 MHz, $CDCl_3$, δ): 8.43-8.28 (m, 1H), 8.20-8.03 (m, 2H), 7.95-7.60 (m, 13H), 7.58-7.46 (m, 1H), 6.03-5.88 (m, 1H), 4.93 (s, 2H), 4.14-3.90 (m, 2H), 2.51-2.27 (m, 2H). ^{13}C NMR (100 MHz, $CDCl_3$, δ): 162.26, 161.03, 136.43, 134.40, 133.49, 132.51, 121.69, 120.00, 118.15, 115.68, 106.53, 105.71, 103.42, 102.28, 36.71, 30.95, 27.60, 24.07. HRMS: m/z = 407.4734 [M − Br].

Synthesis of M7:

The 1.6 M hexane solution of *n*-BuLi (20.00 mL, 32.08 mmol) was added dropwise to an agitating superdry THF solution (40 mL) of 2-bromoanisole (5 g, 26.73 mmol) at -78 °C under an inert atmosphere of nitrogen. After stirring for 1 h, 4-bromoanisole (5.8 g, 26.73 mmol) was added into the mixture. The reaction was stirred at 25 °C for 12 h. 7 was purified through column chromatography (petroleum ether), yield 77%. In nitrogen atmosphere, 7 (2 g, 5.68 mmol) was added into the mixture of 4-bromo-1-butene (1.53 g, 11.4 mmol) and DMF (20 mL). The reaction was stirred at 120 °C for 24 h. The M7 was purified through column chromatography (methanol : dichloromethane = 20 : 1), yield 80%. ^{1}H NMR (400 MHz, DMSO-*d6*, δ): 7.98-7.58 (m, 6H), 7.29 (m, 6H), 6.01-5.61 (m, 1H), 5.29-5.03 (m, 1H), 4.45-4.04 (m, 1H), 3.51 (m,1H), 3.33 (s, 6H), 2.88 (s, 2H), 2.72 (s, 2H), 2.27 (s, 1H), 1.61 (m, 1H). ^{13}C NMR (100 MHz, DMSO-*d6*, δ): 164.41, 162.88, 135.92, 117.29, 116.32, 110.39, 109.59, 56.80, 36.55, 31.29, 26.01, 21.16, 18.73. HRMS: m/z = 407.4734 [M − Br].

Synthesis of P7 and P8:

The polymer was prepared by copolymerization of the M6 monomer (100 mg, 0.24 mmol, 1 eq) and acrylamide (872.23 mg, 12.28 mmol, 50 eq) by a radial polymerization with 2,2'-azobis(2-methylpropionitrile) (AIBN) (40 mg) as radical initiator at 65°C under an argon atmosphere in DMF for 12 h. The resulting mixture was added into methanol to precipitate polymeric materials. Precipitation was repeatedly washed with methanol to give purified polymers. P7 polymer was prepared by using the same method.

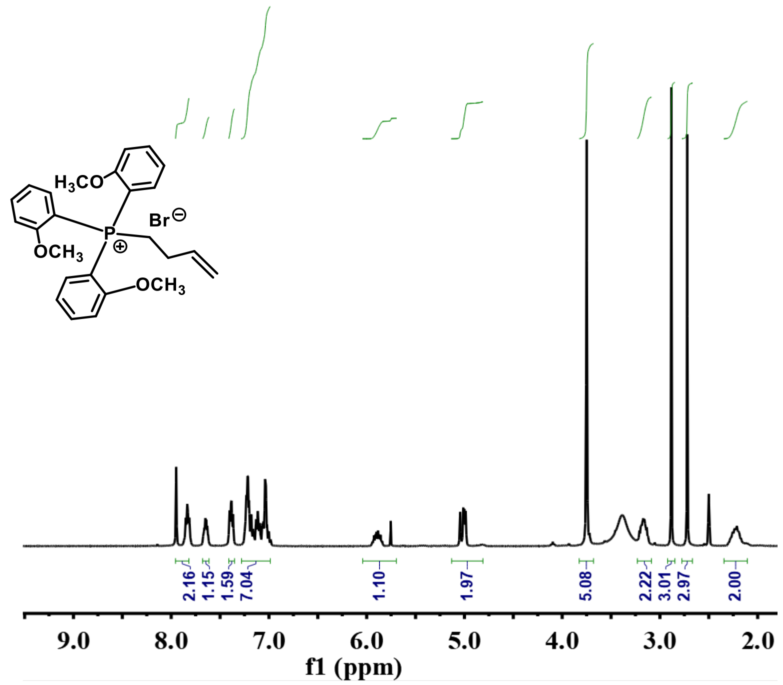

Supplementary Fig. 18 ^{1}H NMR spectrum of M6 in DMSO-d_6.

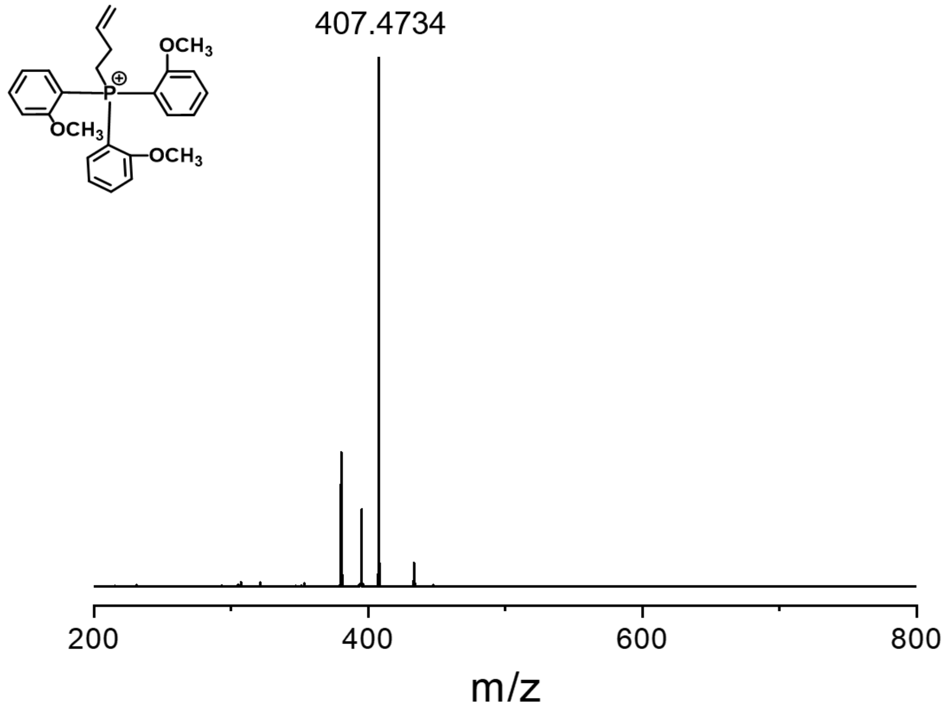

Supplementary Fig. 19 HRMS spectrum of M6.

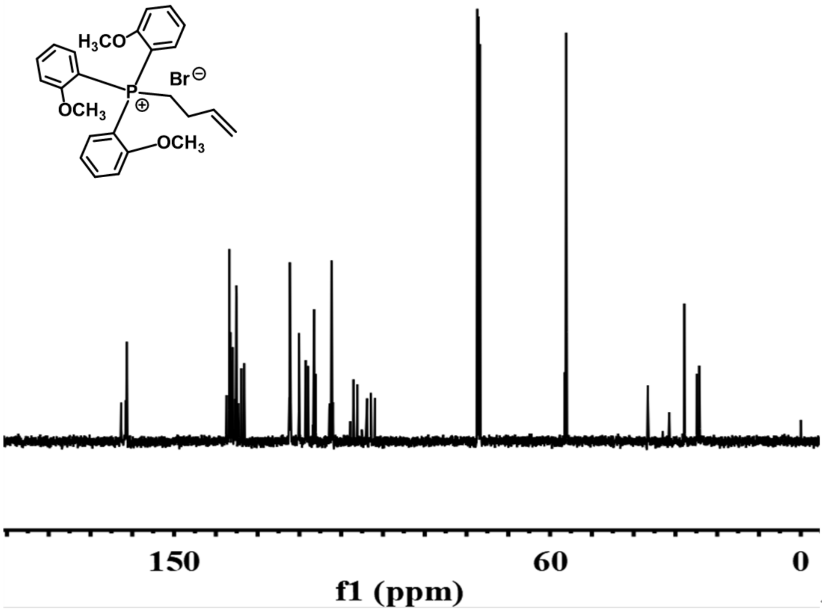

Supplementary Fig. 20 ^{13}C NMR spectrum of M6 in $CDCl_3$.

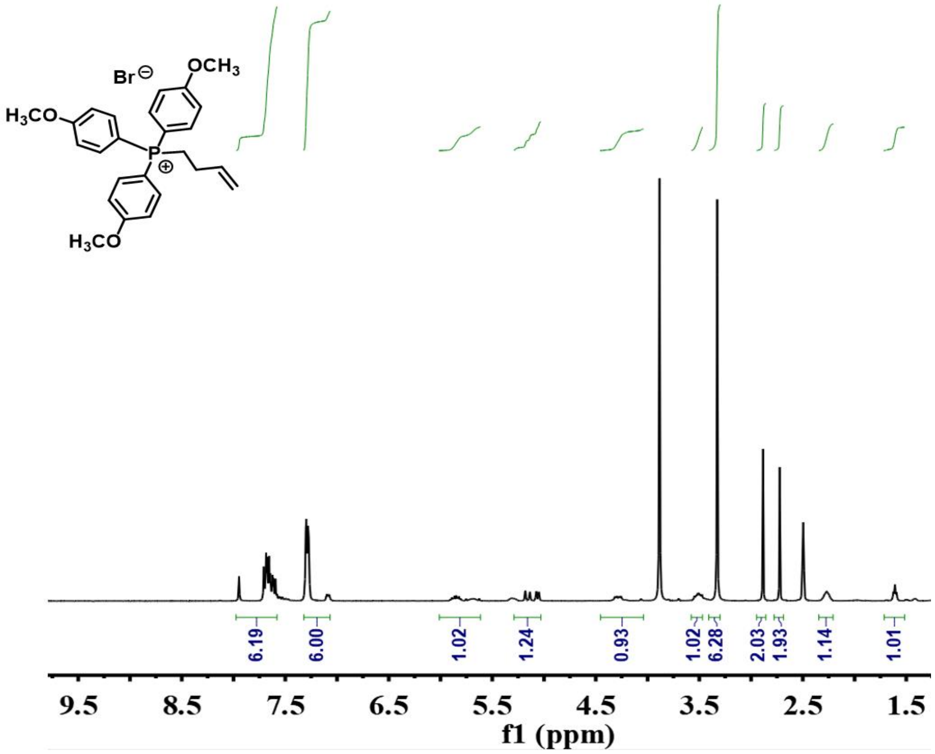

Supplementary Fig. 21 ^{1}H NMR spectrum of M7 in DMSO-d_6.

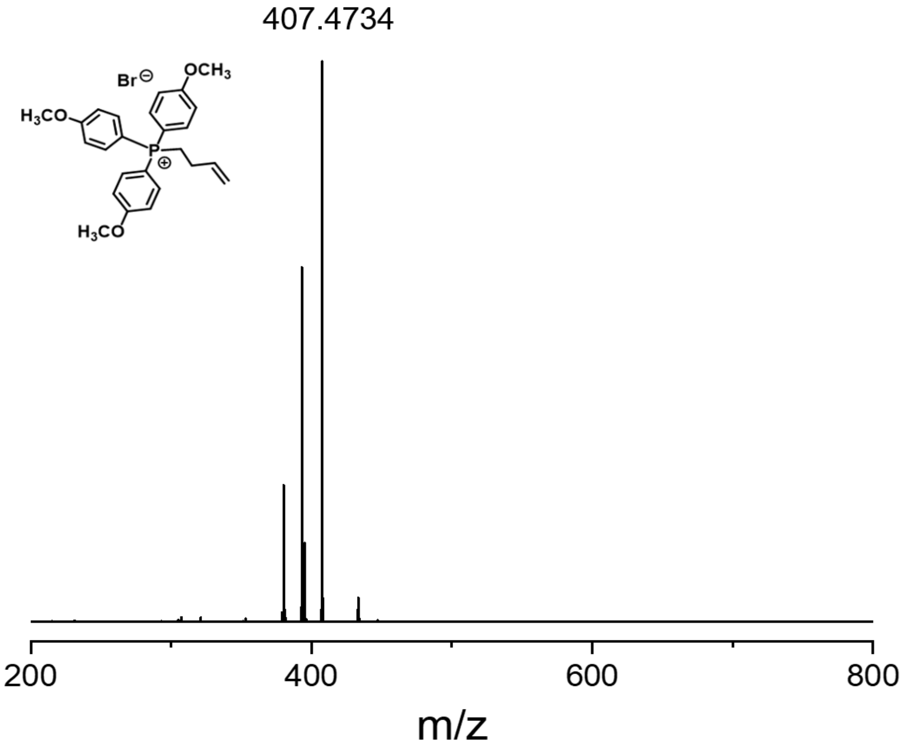

Supplementary Fig. 22 HRMS spectrum of M7.

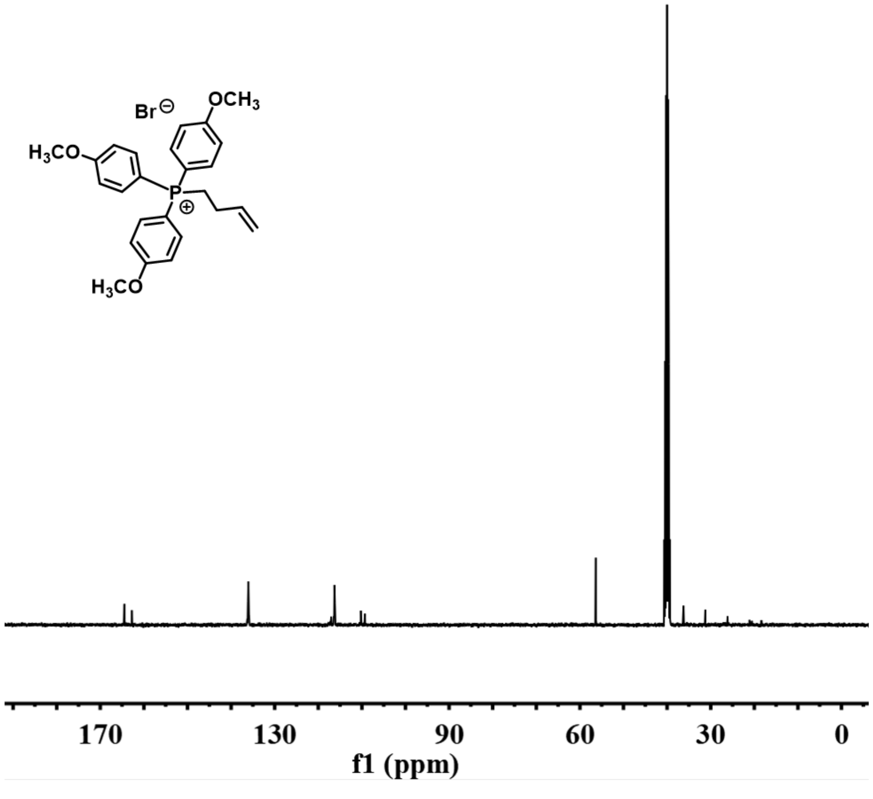

Supplementary Fig. 23 ^{13}C NMR spectrum of M7 in DMSO-d_6.

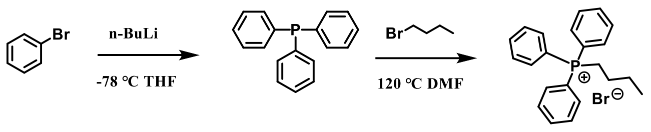

1 C1

Supplementary Fig. 24 Synthesis. Synthetic route of C1.

Synthesis of C1:

The 1.6 M hexane solution of n-BuLi (24.0 mL, 38.46 mmol) was added dropwise to an agitating superdry THF solution (40 mL) of bromobenzene (5.0 g, 32.05 mmol) at -78 °C under an inert atmosphere of nitrogen. After stirring for 1 h, chlorodiphenylphosphine (7.0 g, 32.05 mmol) was added into the mixture. The reaction was stirred at 25 °C for 12 h. 1 was purified through column chromatography (petroleum ether), yield 75%. In nitrogen atmosphere, compound 1 (2 g, 7.62 mmol) was added into the mixture of 1-bromobutane (1.2 g, 9.15 mmol).and DMF (50 mL). The reaction was stirred at 120 °C for 24 h. C1 was purified through column chroma-tography (methanol : dichloromethane = 50 : 1), yield 92%. ^{1}H NMR (400 MHz, DMSO-d_6, δ): 7.93-7.87 (m, 3H), 7.84-7.74 (m, 12H), 3.66-3.54 (m, 2H), 1.56-1.41 (m, 4H), 0.88 (m, 3H). ^{13}C NMR (100 MHz, DMSO-d_6, δ): 134.53, 133.53, 129.77, 119.86, 117.92, 23.68, 22.06, 20.25, 13.28.

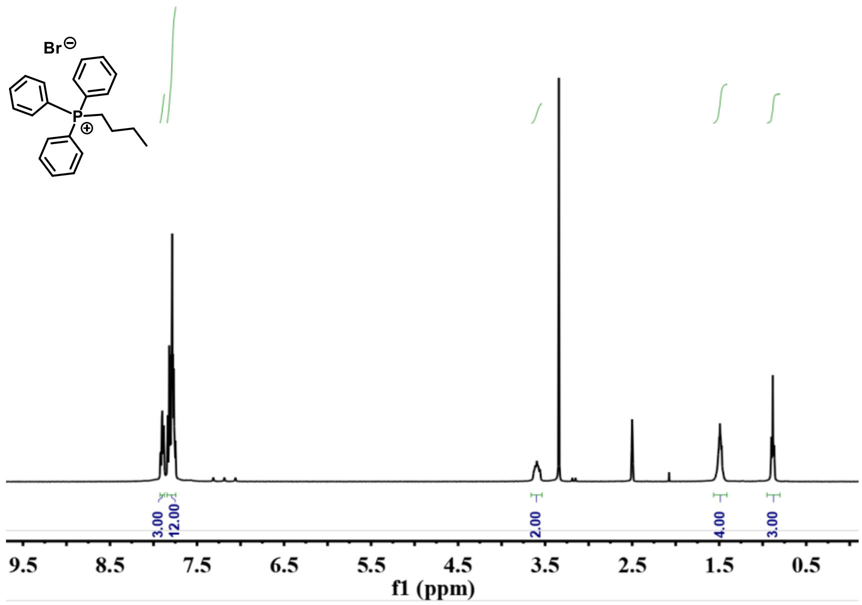

Supplementary Fig. 25 ^{1}H NMR spectrum of C1 in DMSO-d_6.

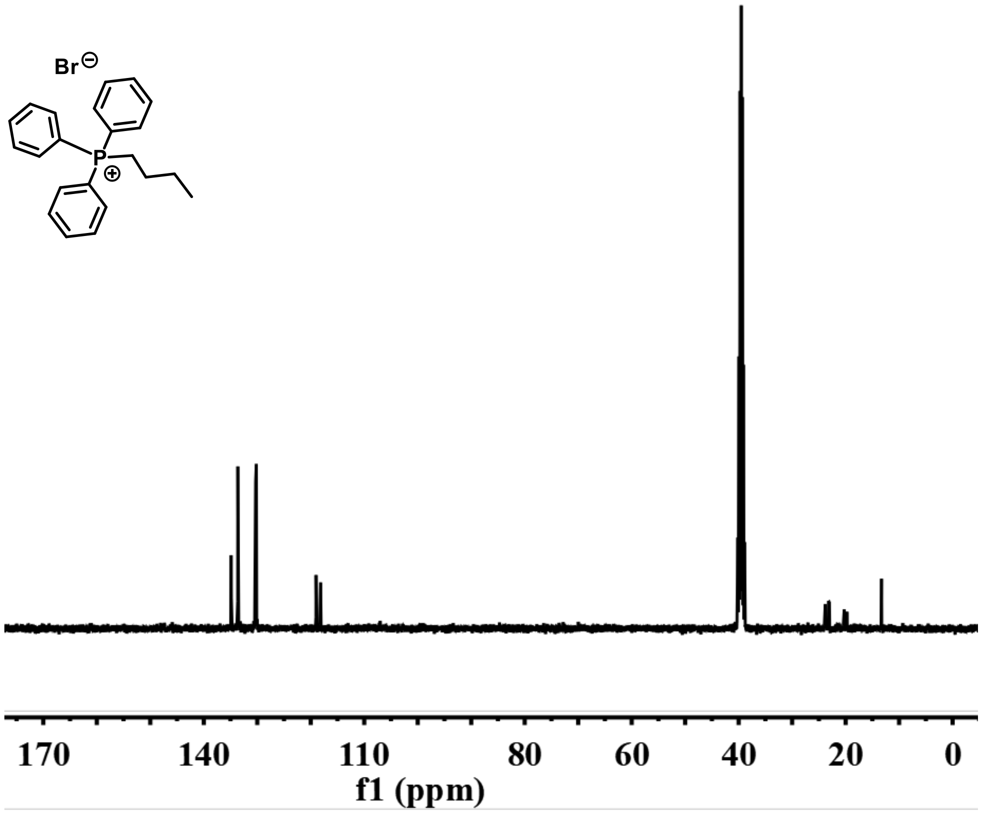

Supplementary Fig. 26 ^{13}C NMR spectrum of C1 in DMSO-d_6.

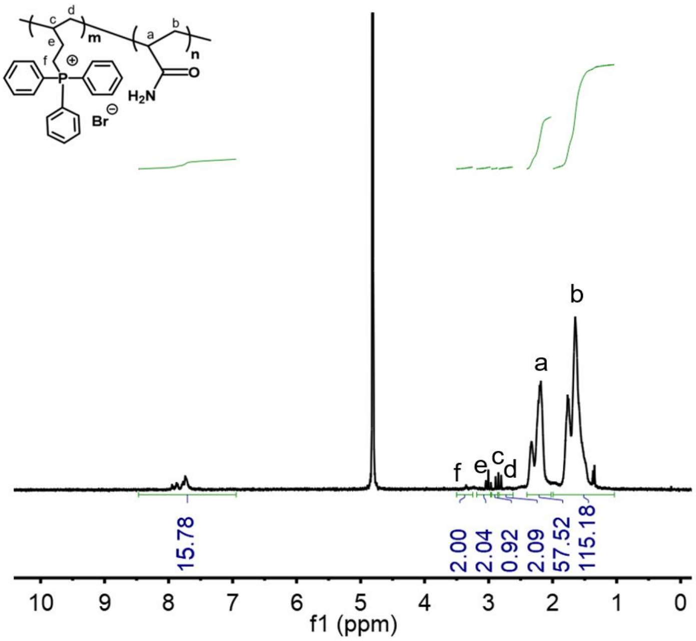

Supplementary Fig. 27 ^{1}H NMR spectrum of P1 in D_2O.

Supplementary Tab. 1 Characterizations of polymers. [a)]

Samples	Mw	Mn	PDI
P1(1/50)	18043	6708	2.6898
P2(1/50)	25569	16015	1.5965
P3(1/50)	28512	19468	1.4645
P4(1/50)	31512	23468	1.3347
P5(1/50)	31324	14670	2.1352
P6(1/50)	29546	18456	1.6001
P7(1/50)	27598	19354	1.4259

a) Mw and Mn were determined by aqueous GPC.

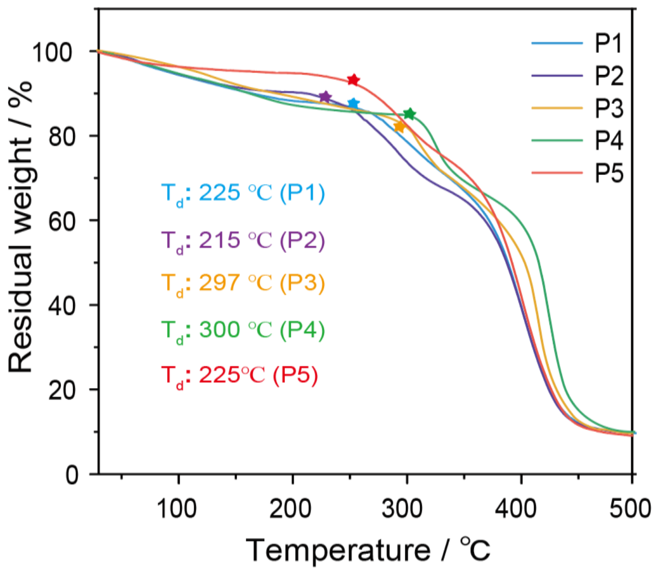

Supplementary Fig. 28 Thermodynamics analysis of polymers. TGA curves of P1-P5 measured in nitrogen atmosphere at a heating rate of 10 °C/min.

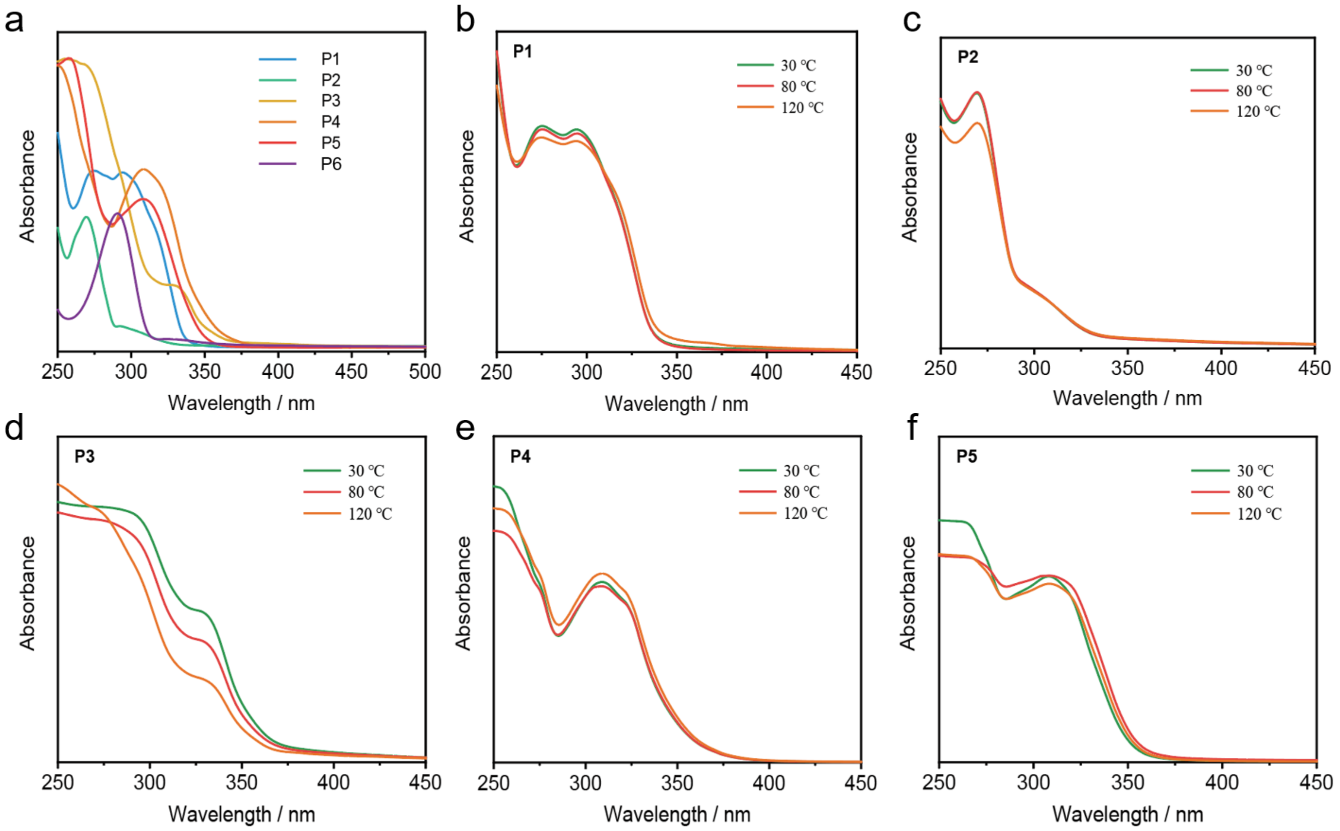

Supplementary Fig. 29 UV absorption of polymers. a The UV absorption spectra of P1-P6 at room temperatures. **b-f** The UV absorption spectra of P1-P5 at 30 ℃, 80 ℃, and 120 ℃, respectively.

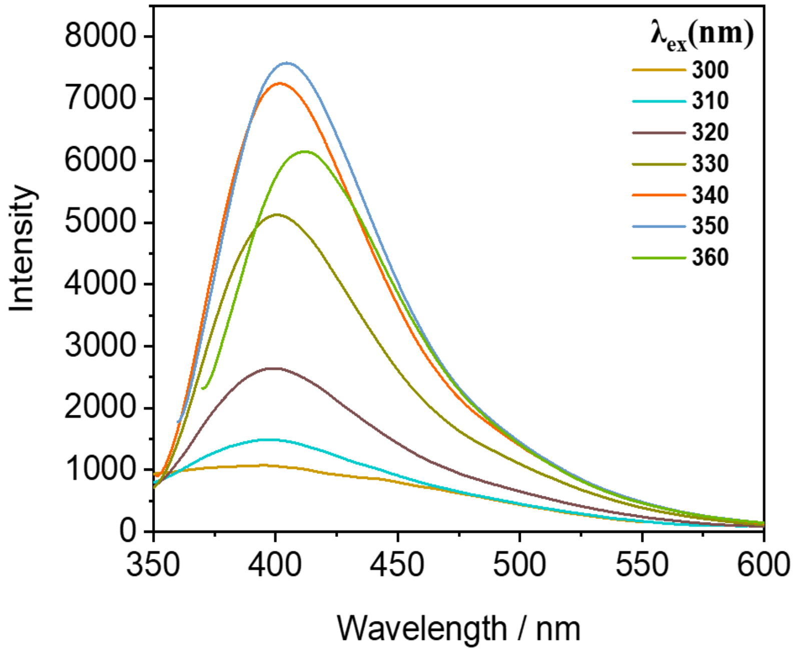

Supplementary Fig. 30 PL spectra of polymers P1. The PL spectra of polymers P1(1/50) in the solid state at various excitation wavelengths (300-360 nm).

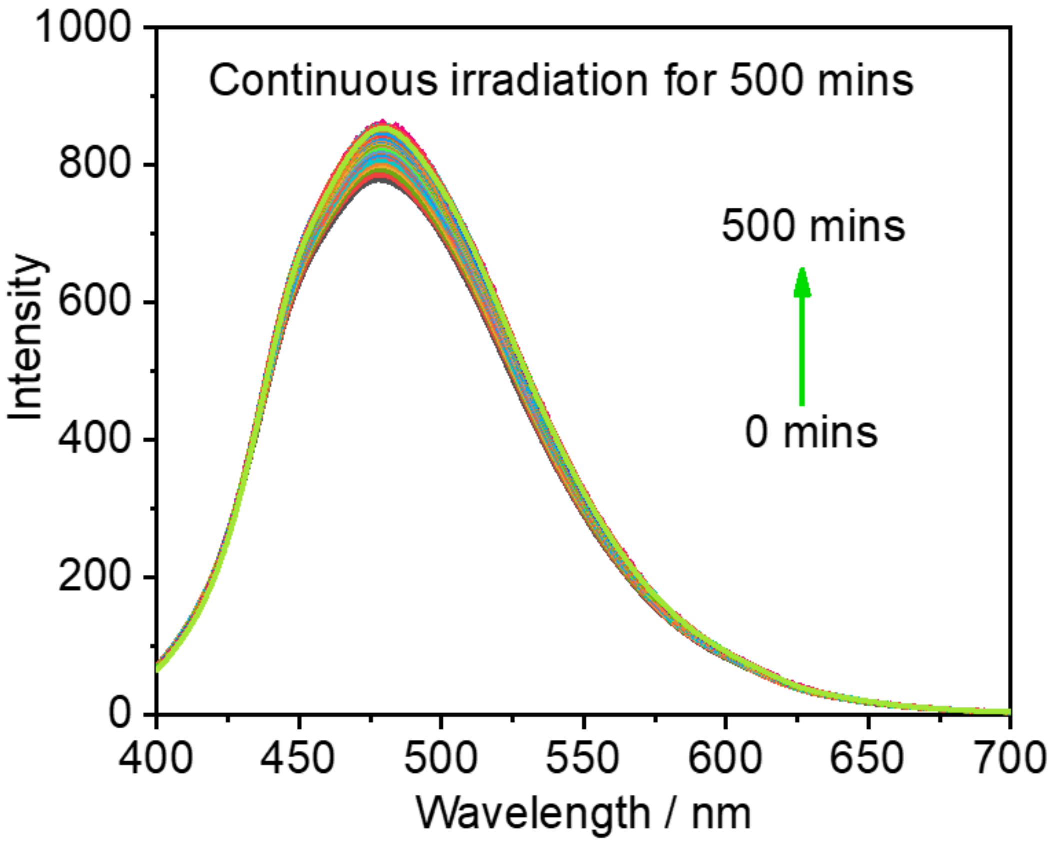

Supplementary Fig. 31 The photostability of P1. The delayed PL spectral changes of **P1** in solid state under continuous UV irradiation at 300 nm for 500 mins.

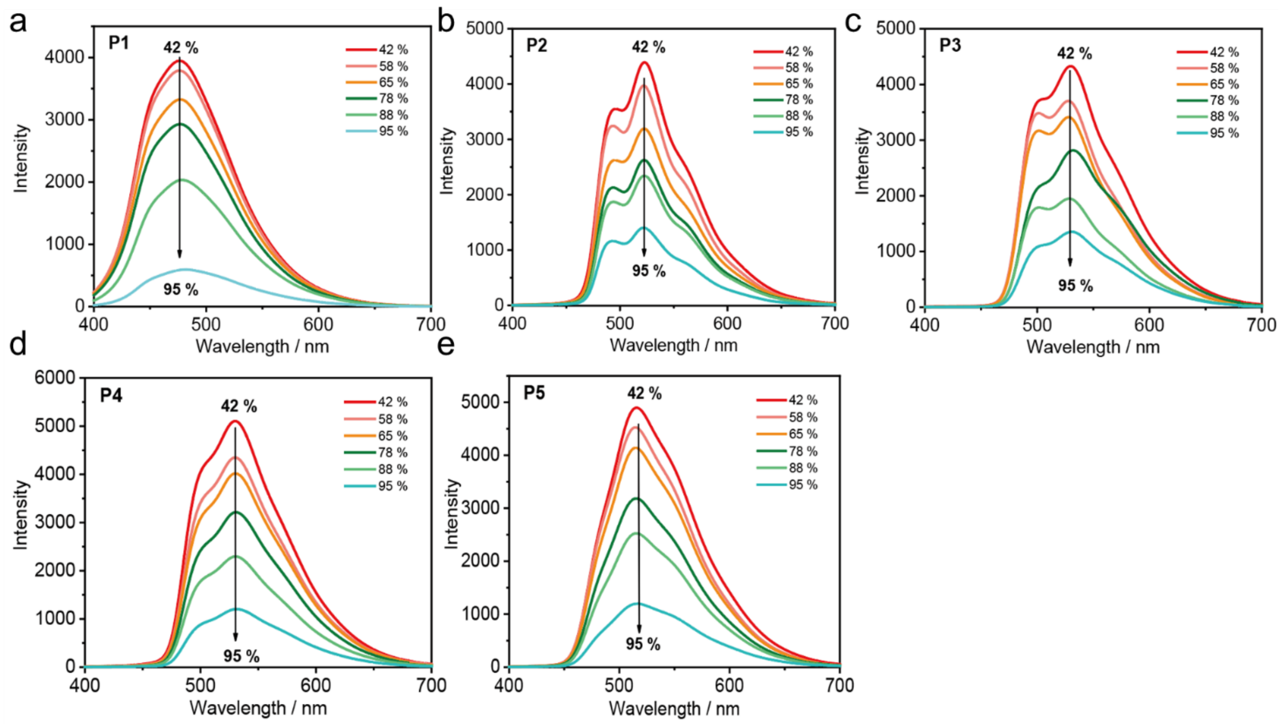

Supplementary Fig. 32 RTP of P1-P5 change under different humidity conditions. a-e The delayed PL spectra spectral changes of P1-P5 at various RHs

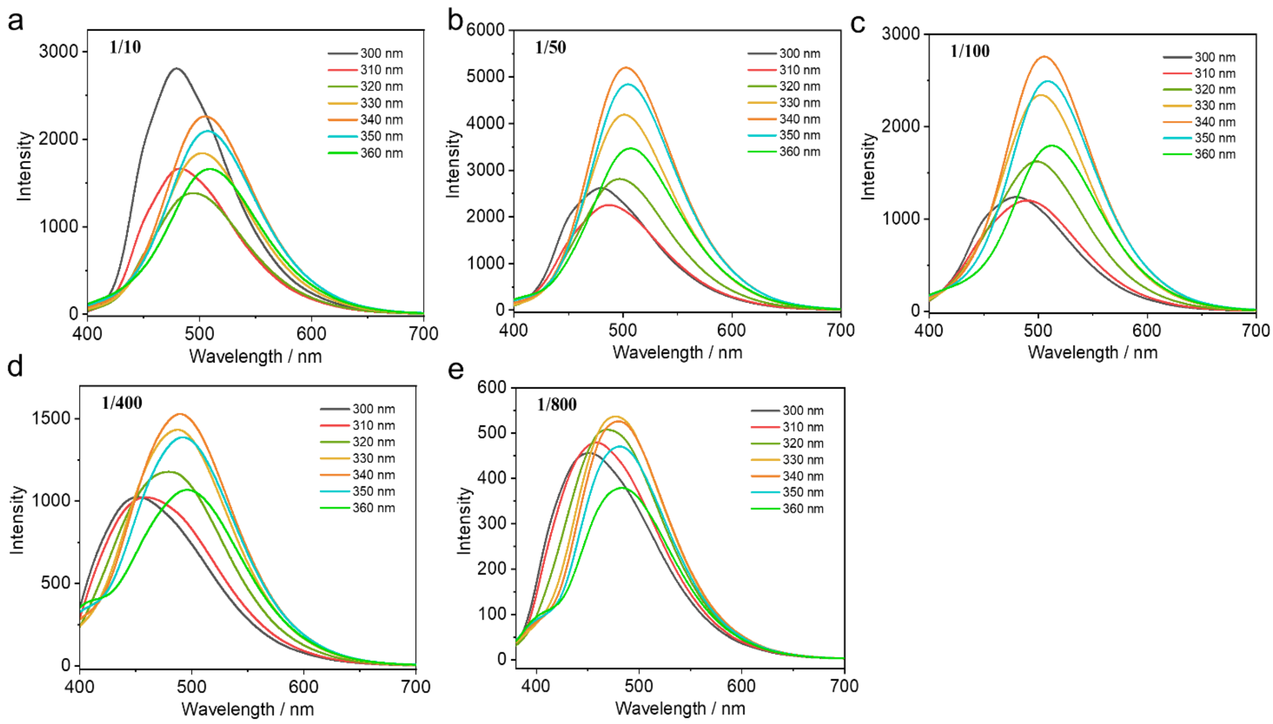

Supplementary Fig. 33 The delayed PL spectra of P1(1/10-1/800). a-e The delayed PL spectra of **P1** (1/10-1/800) at various excitation wavelengths (300-360 nm).

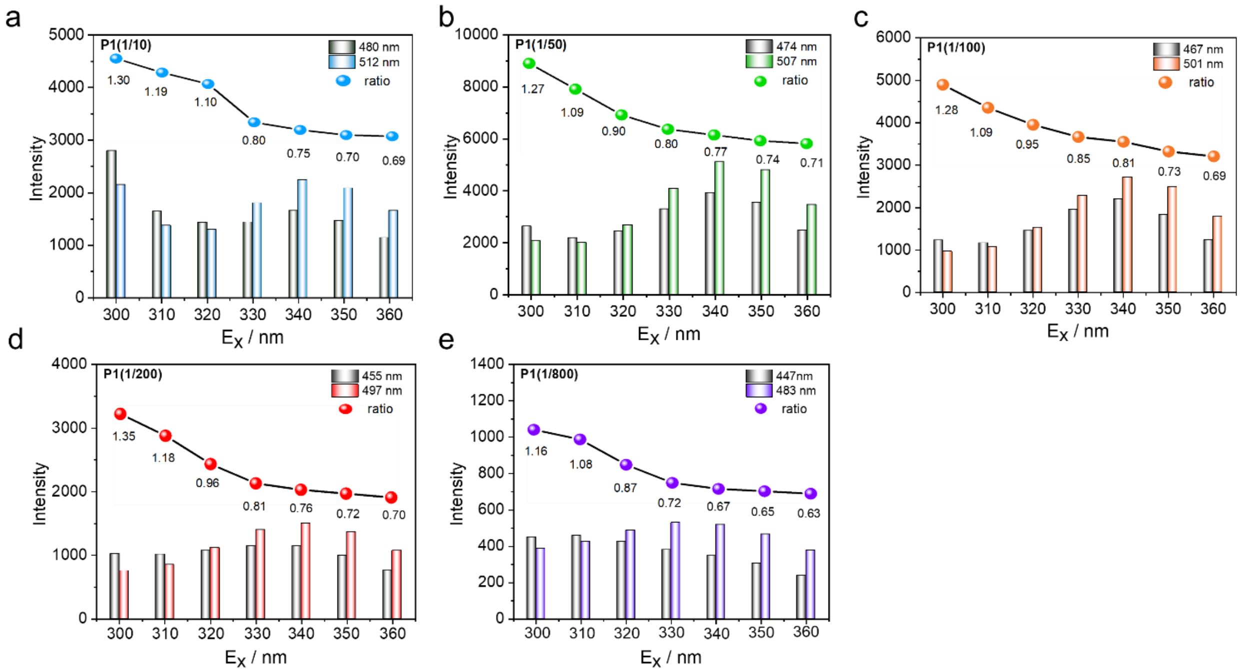

Supplementary Fig. 34 P1(1/10-1/800) excited at different wavelengths. a-e The ratiometric change of two emission wavelengths at various excitation wavelengths (300-360 nm).

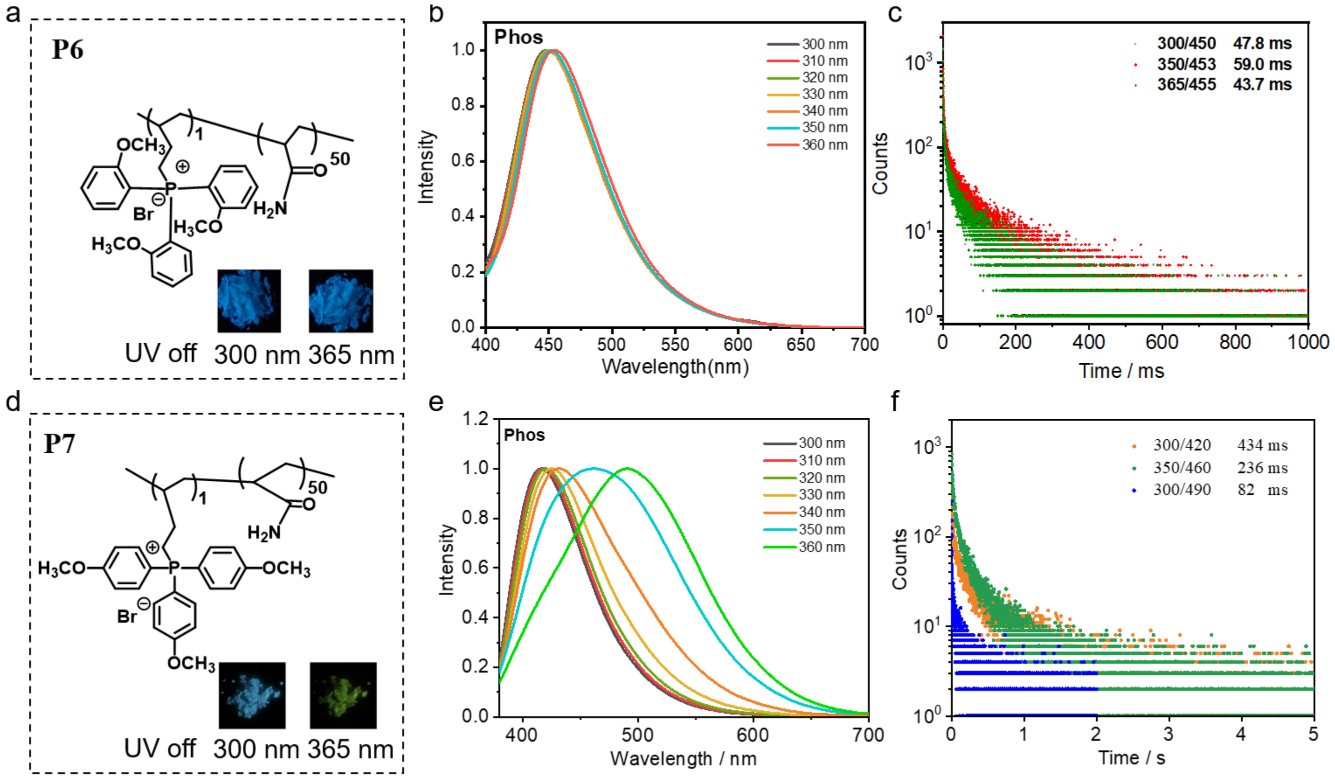

Supplementary Fig. 35 Photophysical properties of P6 and P7. a Chemical structure of P6 and its afterglow photos. **b** Ex-De RTP spectra of P6. **c** Lifetime decay curves of P6 in the solid state. (d) Chemical structure of P7 and its afterglow photos. **e** Ex-De RTP spectra of P7. **f** Lifetime decay curves of P7 in the solid state.

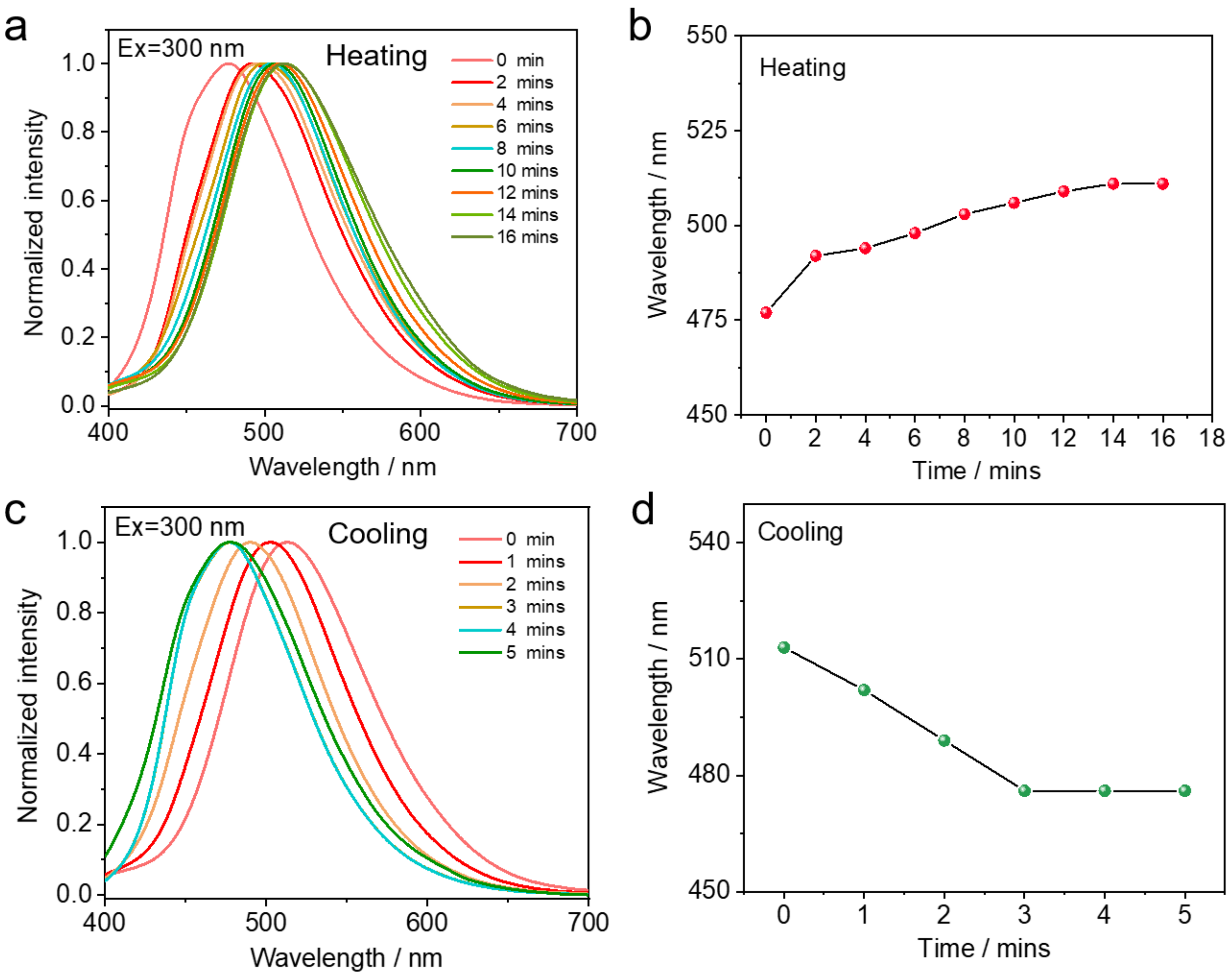

Supplementary Fig. 36 Reversibility of P1 under different temperatures. a, b The phosphorescence spectral changes of P1 upon heating at 120 °C. **c, d** The phosphorescence spectral changes of P1 after removing the heating source.

Supplementary Tab. 2 Photophysical properties of P1 in the solid state under ambient conditions. [a)]

Samples	λ_{ex} [nm]	λ_{em} [nm]	T_1 (ms)	A_1 (%)	T_2 (ms)	A_2 (%)	T_{ave} (ms)
1/10	340	479	32	40	449	60	282
1/50		479	84	13	960	87	846
1/100		479	41	20	751	80	612
1/200		479	344	100			344
1/400		465	6	33	136	67	93
1/800		457	54	100			54

a) λ_{ex} = excitation wavelength of delayed emission; λ_{em} = emission of delayed emission.

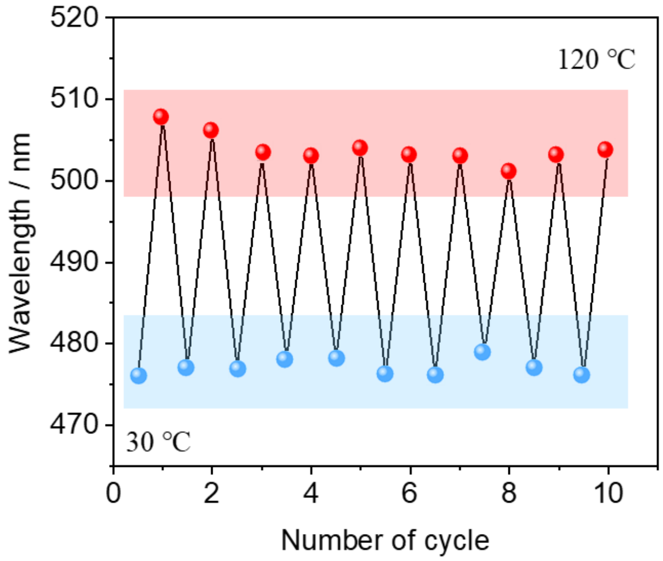

Supplementary Fig. 37 The reversibility of phosphorescence peak between room temperature and high temperature. Ten cycles of phosphorescence wavelength variations measured at 30 °C and 120 °C (**P1**).

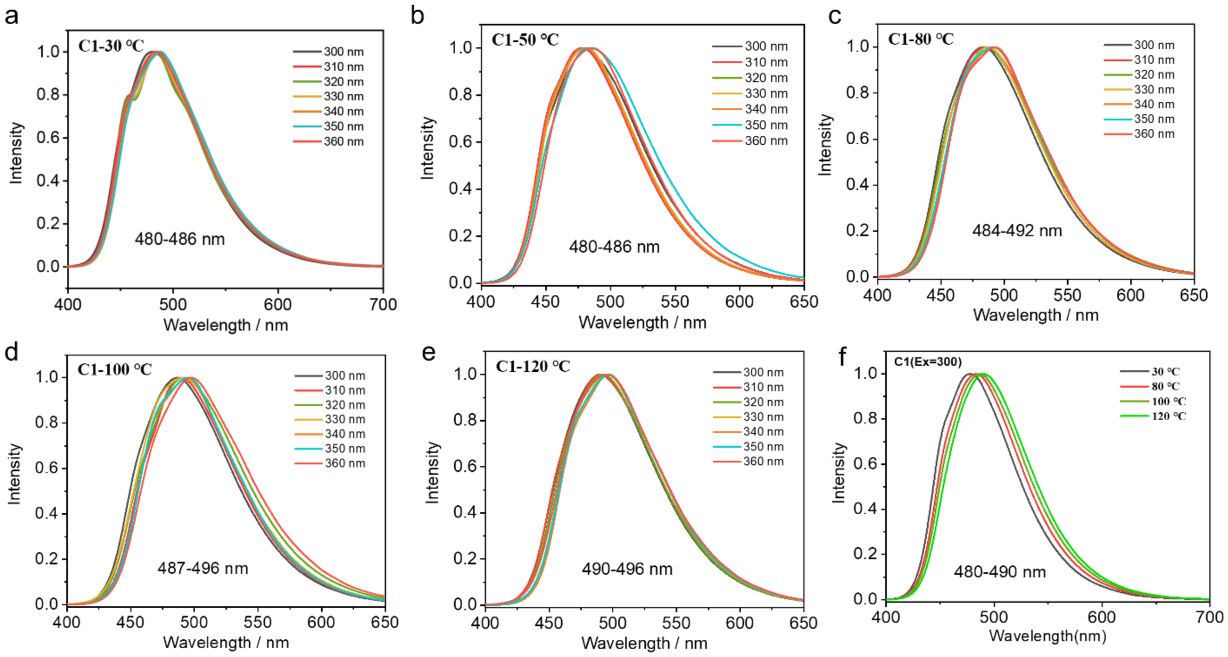

Supplementary Fig. 38 The delayed PL spectra of C1. a-e The delayed PL spectra of C1 at various excitation wavelengths (300-360 nm), upon heating from 30 ℃ to 120 ℃, **f** The delayed PL spectra of C1 upon heating from 30 ℃ to 120 ℃ under 300 nm excitation.

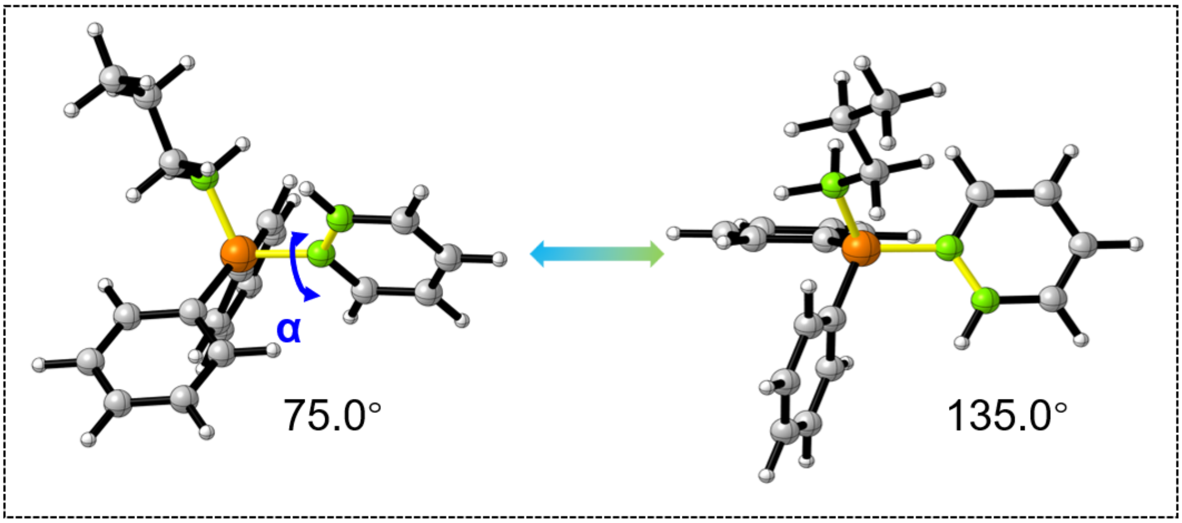

Supplementary Fig. 39 Theoretical calculation. Definition of the dihedral angle (α) between the phenyl ring and P-C planes.

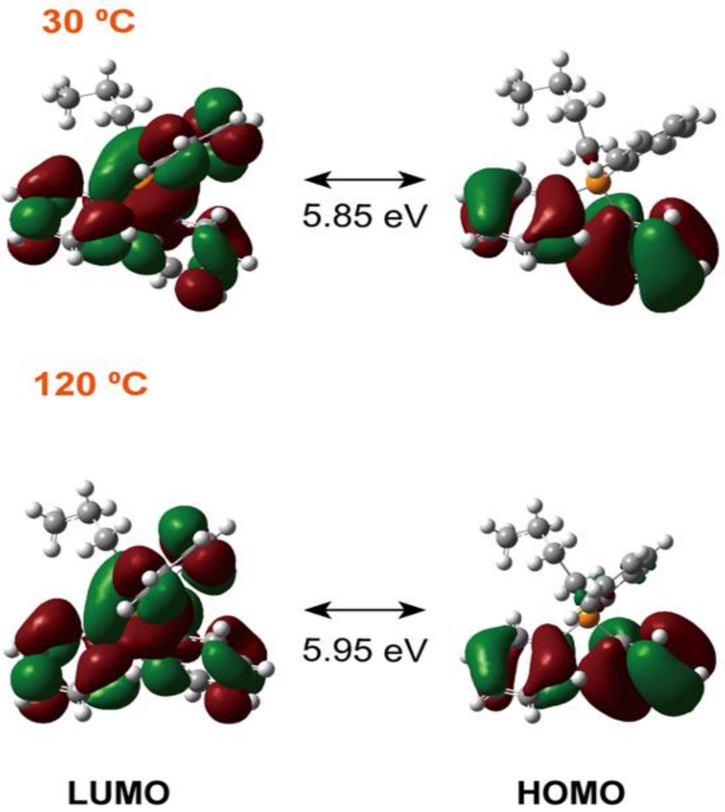

Supplementary Fig. 40 Theoretical calculation. Frontier molecular orbitals and excitation energies of the lowest excited triplet states of different C1 form molecules at TDA B3LYP/def2-SVP level of theory.

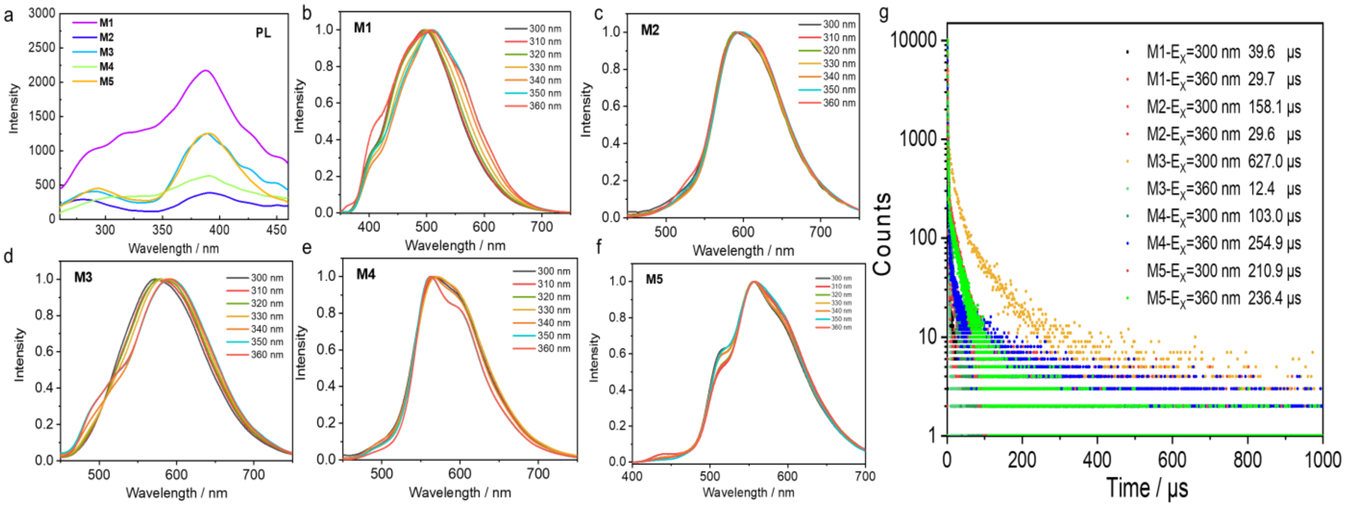

Supplementary Fig. 41 Photophysical properties of M1-M5. a The PL spectra of M1-M5 in the crystal state at 340 nm excitation wavelength. **b-f** The delayed PL spectra of M1-M5 in the crystal state at various excitation wavelengths (300-360 nm). **g** Lifetime decay curves of M1-M5 in the crystal state at room temperature , excitation wavelengths under 300/360 nm.

The prompt emission spectra of M1-M5 in the crystalline state exhibit a weak emission band in the 280-320 nm range and a main band at around 380 nm. The delayed PL spectra indicate their phosphorescence peaks were located in the range of 480-550 nm, and their emission decay time ranges from 12.4 μs to 627.0 μs. Notably, upon changing the excitation wavelengths from 300 to 360 nm, no obvious changes in phosphorescence peaks of these monomers were observed.

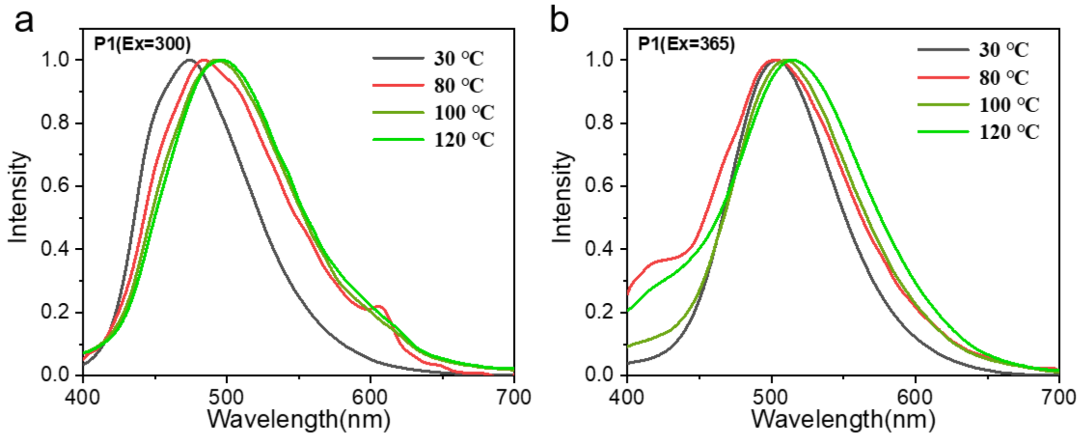

Supplementary Fig. 42 Phosphorescence spectra of P1 at different temperature. a The phosphorescence spectra of P1 upon heating from 30 ℃ to 120 ℃ under 300 nm excitation, **b** The phosphorescence spectra of P1 upon heating from 30 ℃ to 120 ℃ under 365 nm excitation.

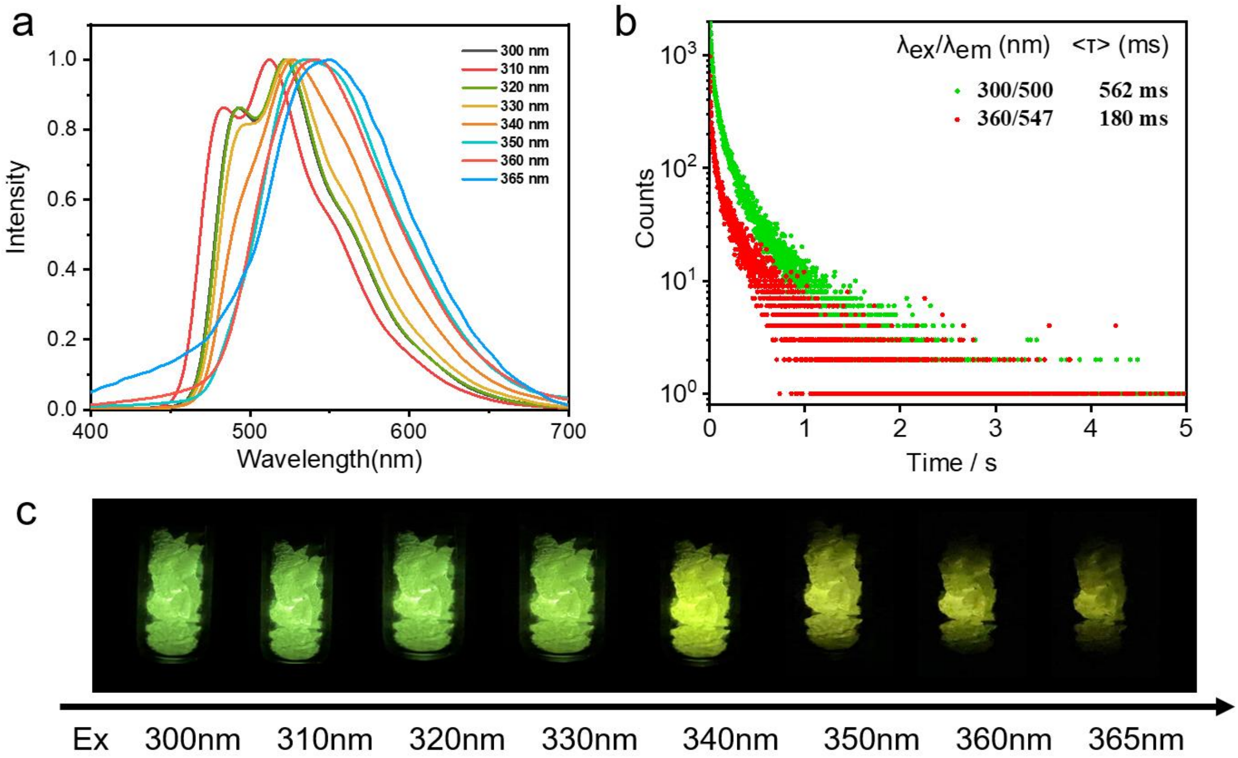

Supplementary Fig. 43 Photophysical properties of P2. a The delayed PL spectra of polymers P2 in the solid state at various excitation wavelengths (300-365 nm). **b** Lifetime decay curves of P2 in the solid state, excitation wavelengths under 300/365 nm. **c** Photographs of P2 under different excitation wavelengths, taken after removing UV irradiation.

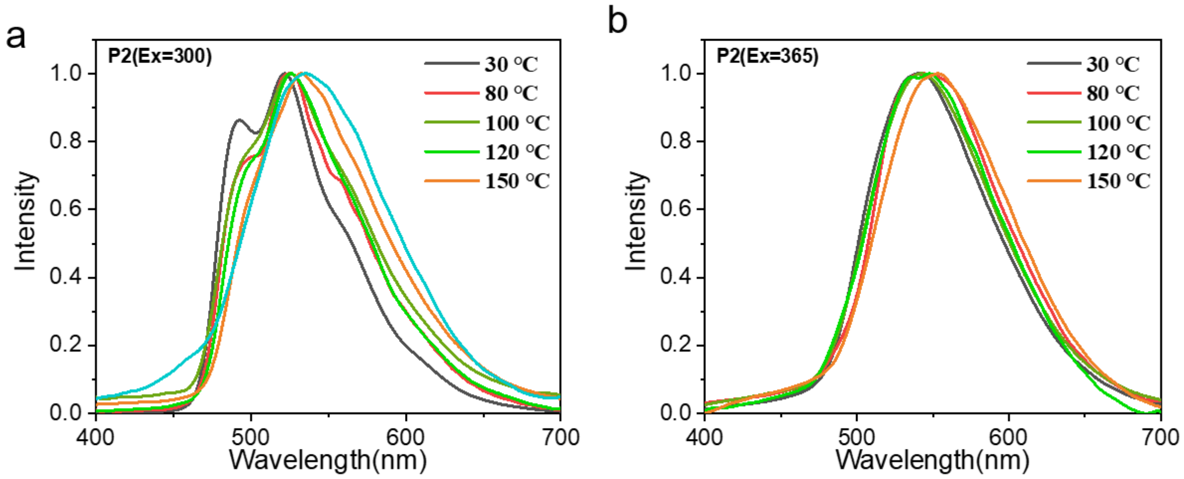

Supplementary Fig. 44 Phosphorescence spectra of P2 at different temperature. a The phosphorescence spectra of P2 upon heating from 30 °C to 150 °C under 300 nm excitation, **b** The phosphorescence spectra of P2 upon heating from 30 °C to 150 °C under 365 nm excitation.

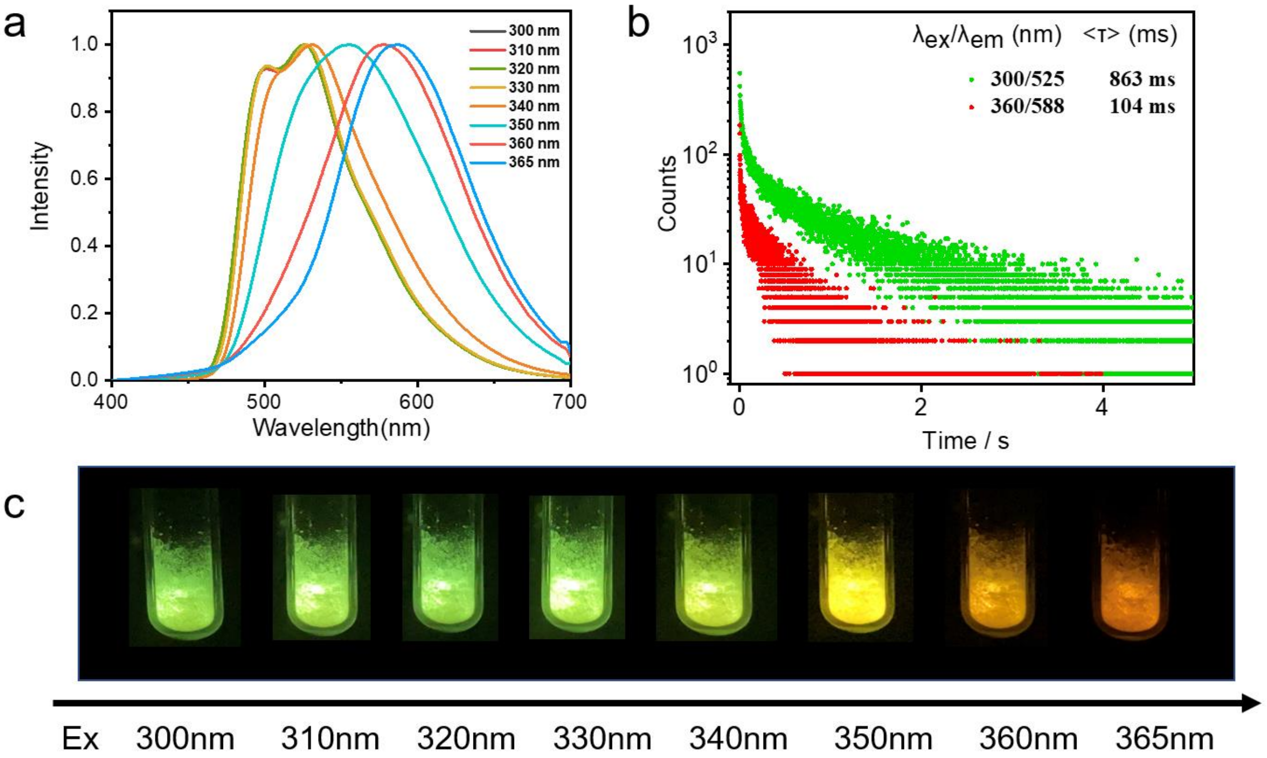

Supplementary Fig. 45 Photophysical properties of P3. a The delayed PL spectra of polymers P3 in the solid state at various excitation wavelengths (300-365 nm). **b** Lifetime decay curves of P3 in the solid state, excitation wavelengths under 300/365 nm. **c** Photographs of P3 under different excitation wavelengths, taken after removing UV irradiation.

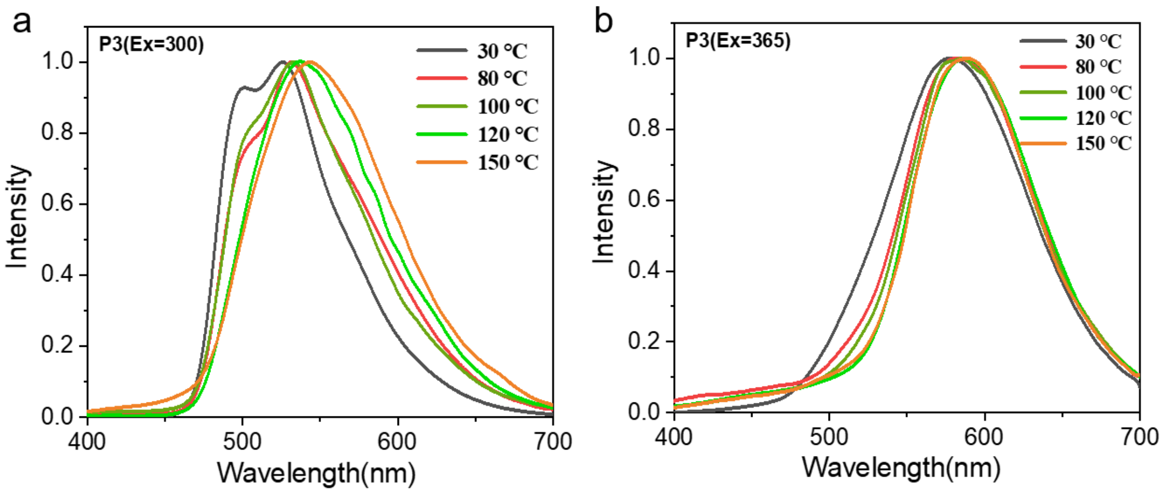

Supplementary Fig. 46 Phosphorescence spectra of P3 at different temperature. a The phosphorescence spectra of P3 upon heating from 30 ℃ to 150 ℃ under 300 nm excitation, **b** The phosphorescence spectra of P3 upon heating from 30 ℃ to 150 ℃ under 365 nm excitation.

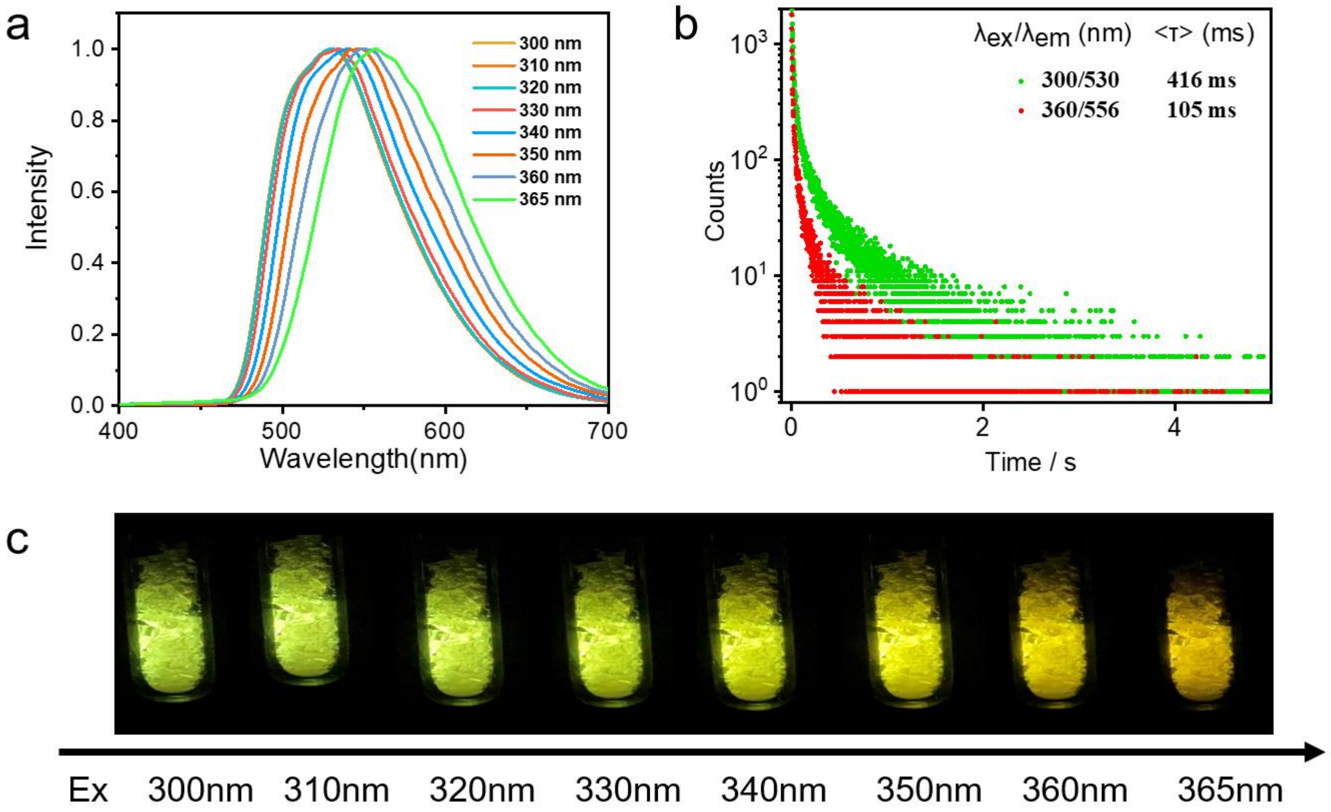

Supplementary Fig. 47 Photophysical properties of P4. a The delayed PL spectra of polymers P4 in the solid state at various excitation wavelengths (300-365 nm). **b** Lifetime decay curves of P4 in the solid state, excitation wavelengths under 300/365 nm. **c** Photographs of P4 under different excitation wavelengths, taken after removing UV irradiation.

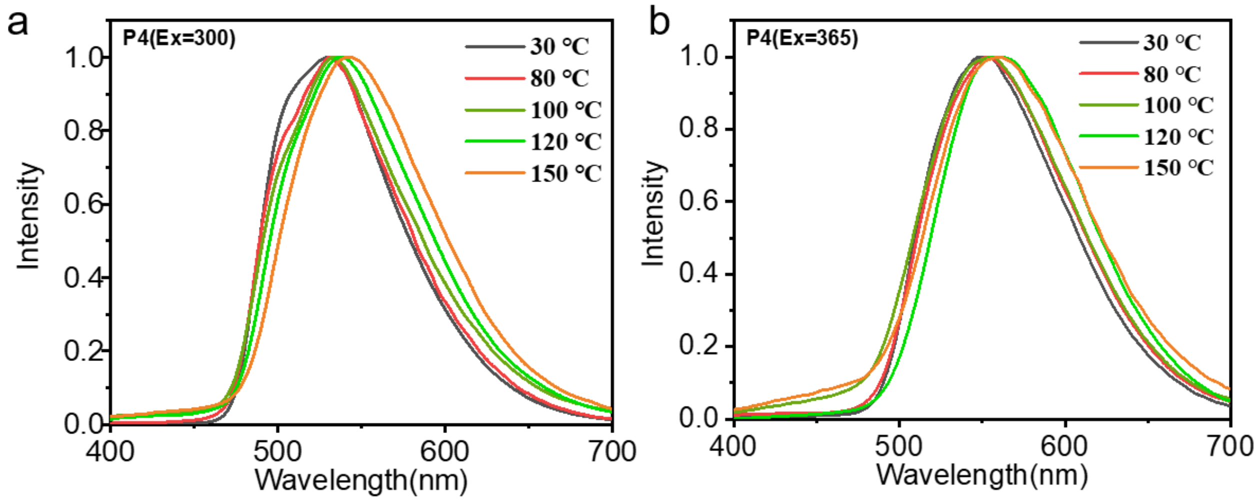

Supplementary Fig. 48 Phosphorescence spectra of P4 at different temperature. a The phosphorescence spectra of P4 upon heating from 30 ℃ to 150 ℃ under 300 nm excitation, **b** The phosphorescence spectra of P4 upon heating from 30 ℃ to 150 ℃ under 365 nm excitation.

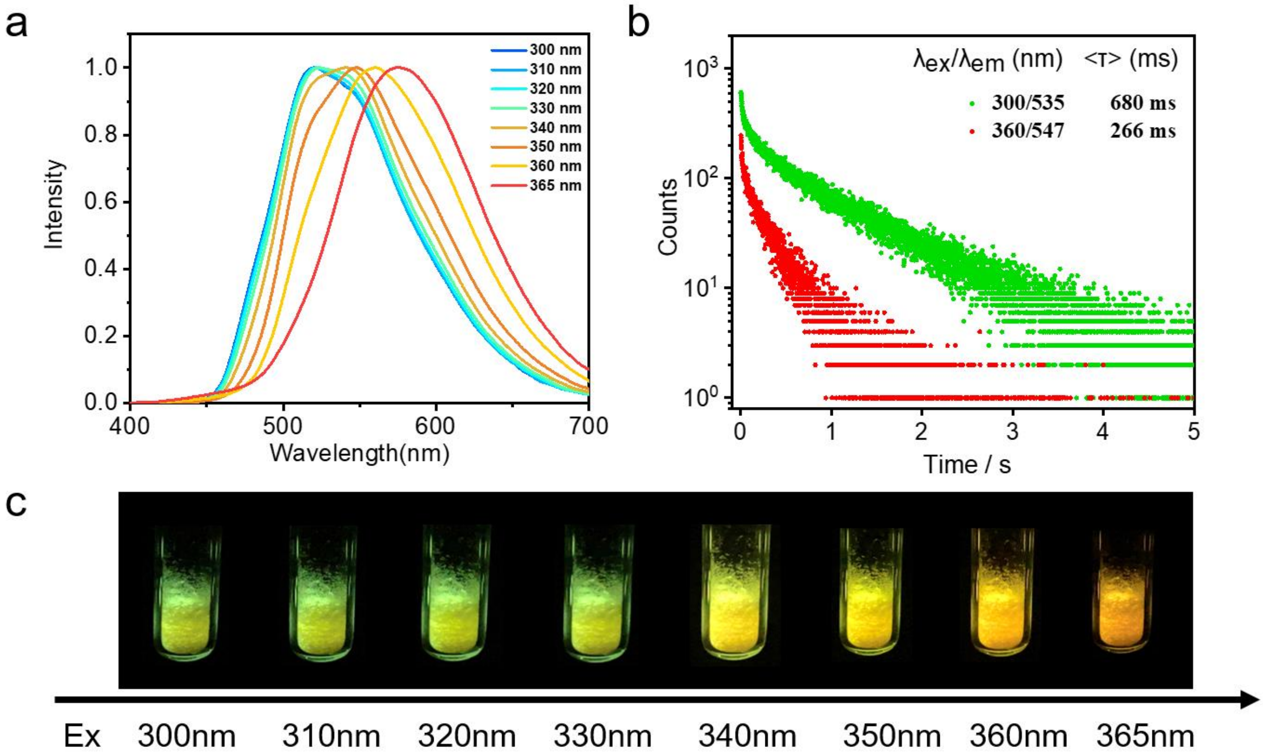

Supplementary Fig. 49 Photophysical properties of P5. a The delayed PL spectra of polymers P5 in the solid state at various excitation wavelengths (300-365 nm). **b** Lifetime decay curves of P5 in the solid state, excitation wavelengths under 300/365 nm. **c** Photographs of P5 under different excitation wavelengths, taken after removing UV irradiation.

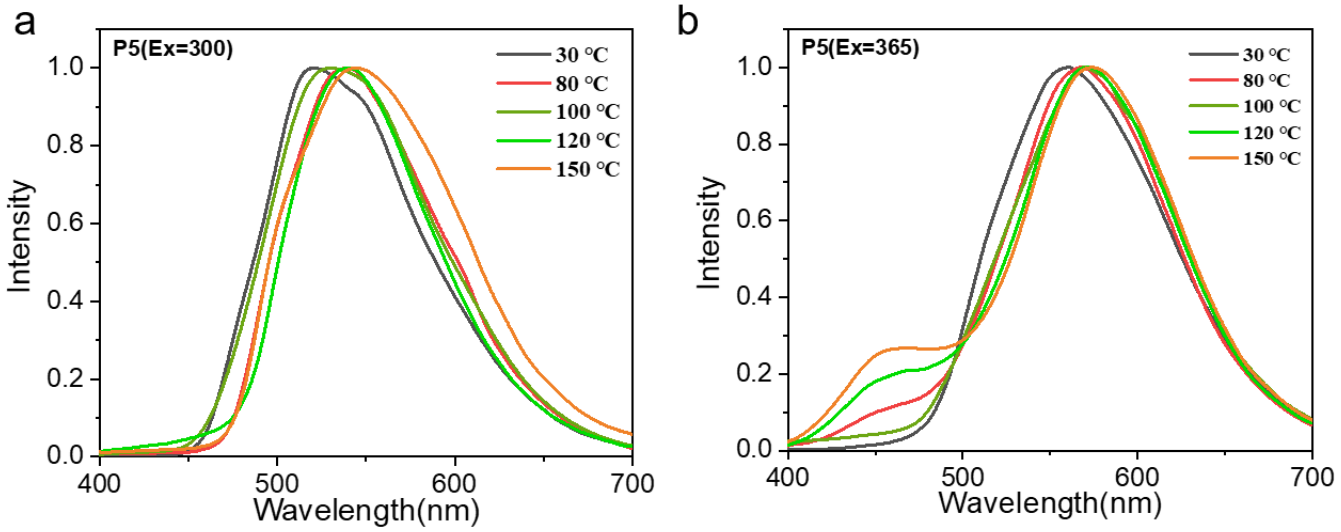

Supplementary Fig. 50 Phosphorescence spectra of P5 at different temperature. a The phosphorescence spectra of **P5** upon heating from 30 ℃ to 150 ℃ under 300 nm excitation, **b** The phosphorescence spectra of **P5** upon heating from 30 ℃ to 150 ℃ under 365 nm excitation.

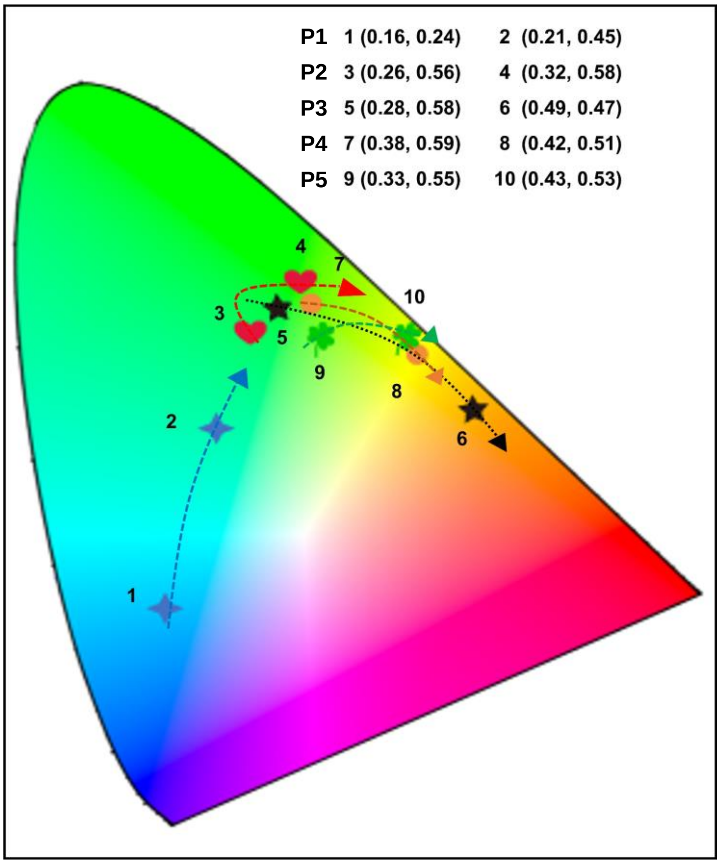

Supplementary Fig. 51 CIE diagram of P1-P5. CIE chromaticity diagram for P1-P5 under 300/365 nm excitation.

Supplementary Tab. 3 Photophysical properties of P1-P5 in the solid state under ambient conditions.[a)]

Samples	λ_{ex} [nm]	λ_{em} [nm]	τ_1 (ms)	A_1 (%)	τ_2 (ms)	A_2 (%)	τ_{ave} (ms)	Φ_F (%)	Φ_P (%)
P1	300	473	79	16	1235	84	1050	1.55	0.56
	365	507	10	8	275	92	261	15.08	0.91
P2	300	500	822	100			562	14.19	5.47
	365	547	34	12	199	88	180	9.41	2.18
P3	300	525	81	9	943	91	863	15.61	8.09
		530	86	10	949	90	833	13.77	7.37
	365	588	104	100			104	18.92	1.46
P4	300	530	84	28	545	72	416	20.64	3.74
	365	556	27	24	128	76	105	21.73	4.89
P5	300	525	84	28	890	72	665	10.31	3.38
	300	535	67	40	1055	60	680	10.77	4.45
	365	630	27	24	341	76	266	17.18	0.68

a) λ_{ex} = excitation wavelength of delayed emission; λ_{em} = emission of delayed emission. Φ_F = fluorescence quantum efficiency; Φ_P = Phosphorescent quantum efficiency.

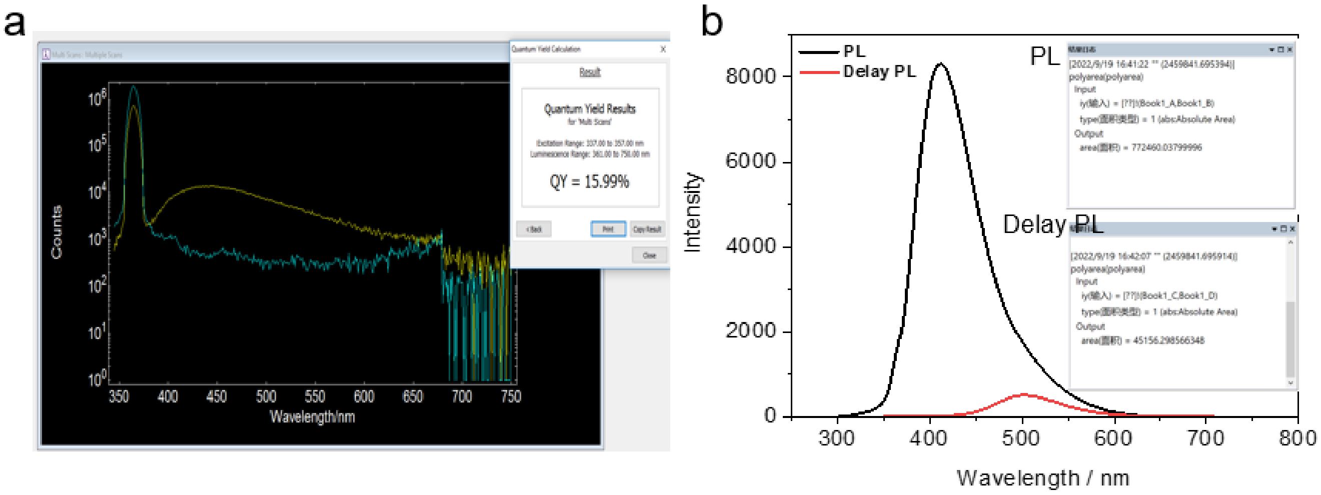

Supplementary Fig. 52 Fluorescence and phosphorescence QY calculation. a The PL spectra of P1 and blank reference PL spectra at 365 nm excitation, **b** The PL spectra and delay PL of P1.

The method to calculate the fluorescence and phosphorescence quantum yields separately was performed according to previous literatures[1,2]. The phosphorescence bands could be obtained in their delayed emission spectrum. According to the structure of phosphorescence bands, the fluorescence and phosphorescence emission bands could be separated in steady state emission spectra. The ratio for fluorescence and phosphorescence quantum yields could be calculated with areas of separated fluorescence and phosphorescence bands. Thus, the fluorescence and phosphorescence quantum yields could be obtained by their total luminescence quantum yields and the ratio for the two relative quantum yields. The calculation method was added in the revised supplementary information.

Photoluminescence quantum efficiency was determined by using Edinburgh FLS980 spectrometer with the integrating sphere (142 mm in diameter) under ambient condition. The fluorescence and phosphorescence quantum efficiency (Φ_F and Φ_P) were calculated through the following formulas:

$$\emptyset_P = \emptyset_E \times \frac{A_P}{A_E} \qquad (1)$$

$$\emptyset_F = \emptyset_E - \emptyset_P \qquad (2)$$

where Φ_E refers to the measured total emission quantum efficiency, AP and AE refer to the integral areas of phosphorescence and photoluminescence components in photoluminescence spectra, respectively.

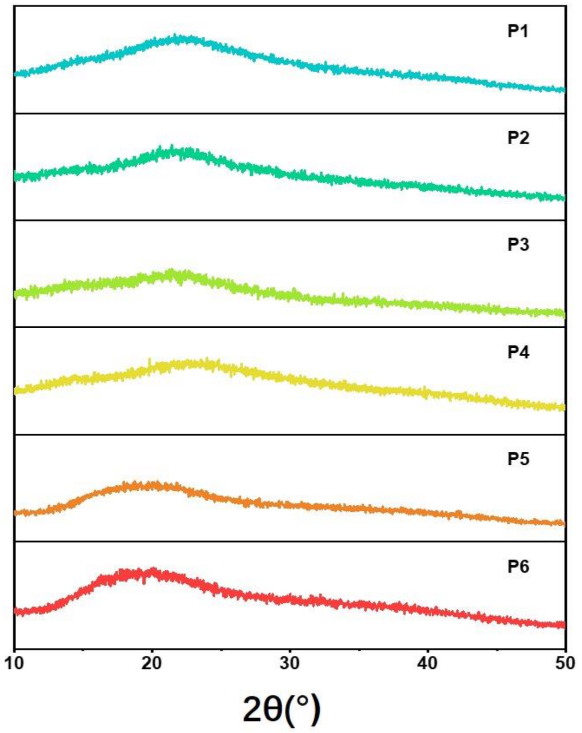

Supplementary Fig. 53 XRD of P1-P6. PXRD pattern of P1-P6.

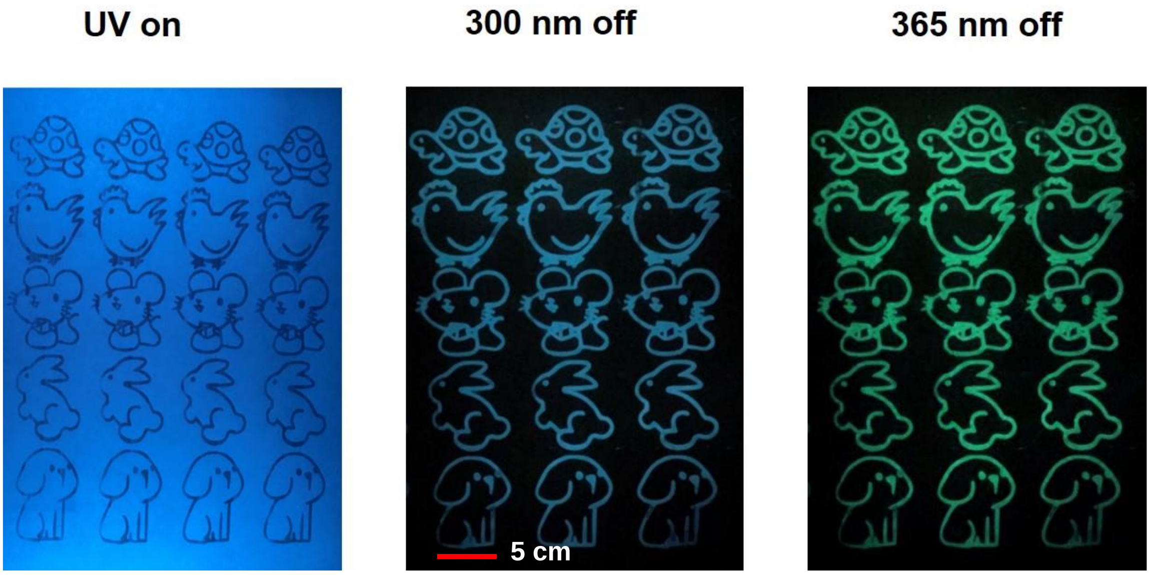

Supplementary Fig. 54. Large area printing applications. Photographs of different animal cartoons printed on a A4 filter paper by using P1 as the ink before and after removing 300 nm and 365 nm irradiation.

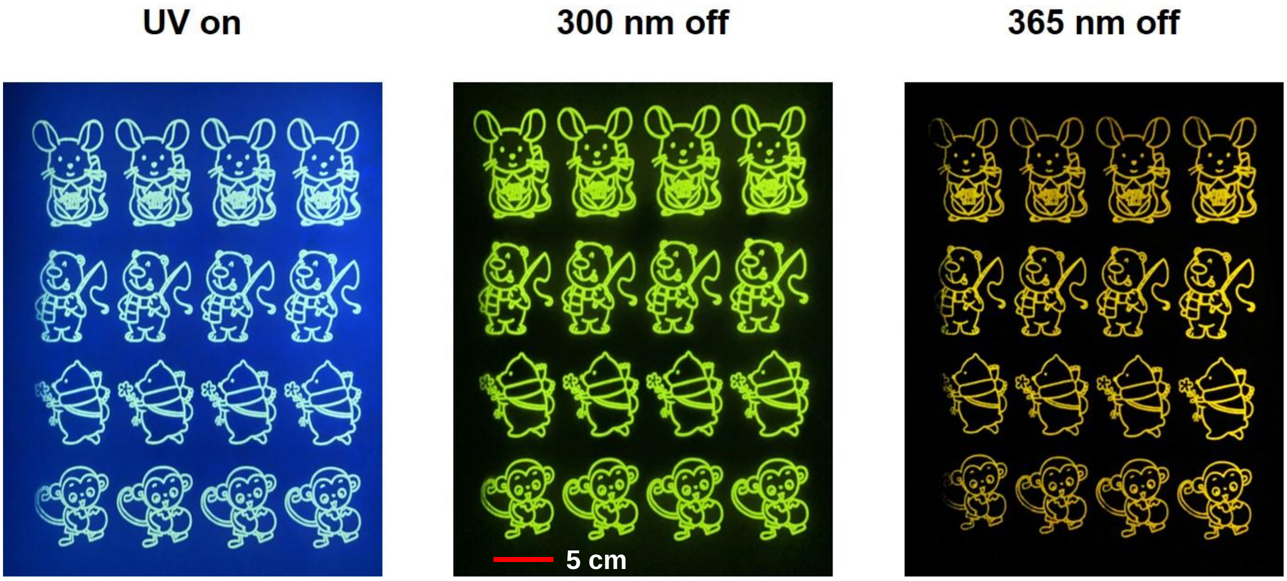

Supplementary Fig. 55. Large area printing applications. Photographs of different animal cartoons printed on a A4 filter paper by using **P3** as the ink before and after removing 300 nm and 365 nm irradiation.

Supplementary References

1. Lei, Y.X. et al. Wide-range color-tunable organic phosphorescence materials for printable and writable security inks. *Angew. Chem. Int. Ed.* **59**, 16054-16060 (2020).
2. Yang Z. et al. Boosting the quantum efficiency of ultralong organic phosphorescence up to 52% via intramolecular halogen bonding, *Angew. Chem. Int. Ed.* **59**, 17451-17455 (2020).
